# Design, synthesis, and characterization of novel 5-ethylsulfonyl-indazole-3-carboxamides as dual VEGFR-2 and EGFR inhibitors: apoptotic antiproliferative and immunomodulatory evaluations

**DOI:** 10.1039/d5ra07017a

**Published:** 2025-12-03

**Authors:** Lamya H. Al-Wahaibi, Shimaa A. Othman, Hesham A. Abou-Zied, Stefan Bräse, Bahaa G. M. Youssif, Safwat M. Rabea

**Affiliations:** a Department of Chemistry, College of Sciences, Princess Nourah bint Abdulrahman University Saudi Arabia; b Pharmaceutical Organic Chemistry Department, Faculty of Pharmacy, Assiut University Assiut 71526 Egypt bgyoussif@ju.edu.sa +20-2-01044353895; c Medicinal Chemistry Department, Faculty of Pharmacy, Deraya University Minia Egypt; d Institute of Biological and Chemical Systems, IBCS-FMS, Karlsruhe Institute of Technology 76131 Karlsruhe Germany braese@kit.edu; e Medicinal Chemistry Department, Faculty of Pharmacy, Minia University Minia 61519 Egypt; f Apogee Pharmaceuticals 4475 Weyburn Dr, Suite 105 Burnaby BC V6V2H8 Canada

## Abstract

This study focused on the design and synthesis of a novel series of 5-ethylsulfonyl-indazole-3-carboxamides (8a–l) as dual inhibitors of VEGFR-2 and EGFR. Compounds 8g and 8h emerged as the most efficient derivatives among all evaluated compounds against breast (MCF-7) and colorectal (HCT-116) cancer cell lines, exhibiting IC_50_ values of 24 and 28 nM for HCT-116 and MCF-7 cell lines, respectively, for 8g, and 23 and 25 nM for the same cell lines for 8h. Compounds 8g and 8h exhibited a promising safety margin against normal cells (WI-38) (IC_50_ values > 150 nM). *In vitro* enzyme assays demonstrated that compounds 8g and 8h exhibited potent inhibition of VEGFR-2 and EGFR. Furthermore, compounds 8g and 8h induced apoptosis by activating Bax, p53, caspase-3, 8, and 9, as well as down-regulating Bcl-2. Compounds 8g and 8h reduced TNF-α and IL-6 levels compared to dexamethasone. The computational investigation of compound 8h, a novel indazole-based urea derivative, was undertaken to rationalize its potent dual inhibition of EGFR and VEGFR-2. Molecular docking studies revealed a high binding affinity and a favorable interaction profile with key kinase residues, particularly hinge-region contacts with Met769 (EGFR) and Glu885/Asp1046 (VEGFR-2). Follow-up molecular dynamics (MD) simulations confirmed the stability of the 8h–EGFR complex over 150 ns, characterized by persistent hydrogen bonding, low RMSF in the binding site, and consistent radius of gyration. Quantum mechanical (QM) analyses, including DFT and MEP mapping, revealed a HOMO–LUMO gap of 4.55 eV, high dipole moment (9.3 D), and distinct electron-rich/hydrogen-bonding regions, supporting strong molecular interactions. Additionally, SwissADME profiling demonstrated acceptable drug-likeness, moderate solubility, and a low CYP-inhibition profile, suggesting favorable pharmacokinetics compared to the reference inhibitor erlotinib. These integrated computational findings align with experimental data on antiproliferative effects and kinase inhibition, reinforcing compound 8h as a promising dual-target anticancer candidate.

## Abstract 2

This study focused on the development of a novel series of 5-ethylsulfonyl-indazole-3-carboxamides (8a–l) as dual inhibitors of VEGFR-2 and EGFR. Compounds 8g and 8h emerged as the most potent derivatives against breast (MCF-7) and colorectal (HCT-116) cancer cell lines.

## Graphical abstract

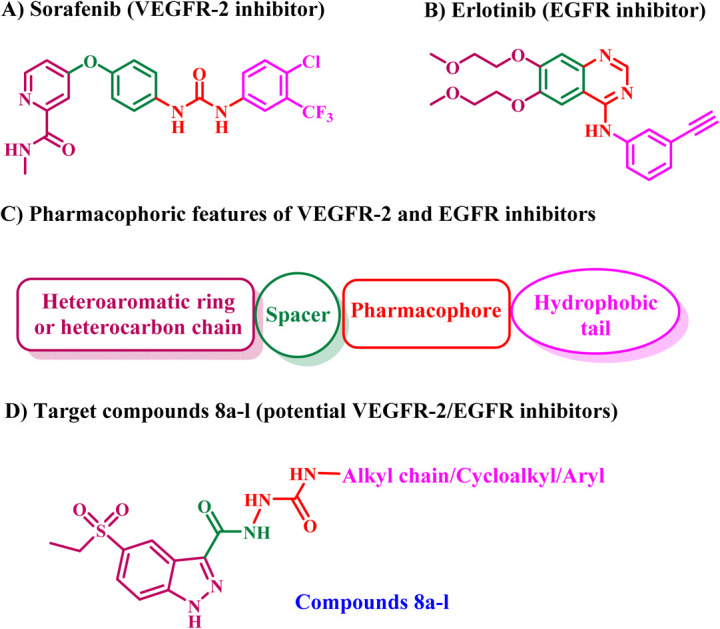

## 1. Introduction

Cancer continues to be a predominant cause of global mortality, accounting for around 10 million deaths in 2020.^1^ The intricate nature of cancer, marked by unregulated cell growth, invasion, and metastasis, poses considerable problems in formulating effective treatments. Targeted therapy has evolved as a highly promising strategy, concentrating on the specific suppression of molecular pathways essential for the survival and proliferation of cancer cells.^2,3^ Protein kinase inhibitors (PKIs) are pivotal to this approach, providing a means of blocking abnormal signaling pathways that promote oncogenesis.^4^

The human receptor tyrosine kinase (RTK) family consists of 58 proteins divided into 20 subfamilies.^5^ These RTKs are crucial for regulating cell proliferation, differentiation, apoptosis, adhesion, and migration.^6–8^ However, hyperactivation of RTKs can trigger the development of various types of cancer. Consequently, the inhibition of RTK activity is increasingly acknowledged as a common strategy in cancer therapy.

The epidermal growth factor receptor (EGFR) and vascular endothelial growth factor receptor (VEGFR-2) are common receptor tyrosine kinases (RTKs).^9^ EGFR regulates a wide range of biological processes, including cell survival, proliferation, and migration.^10^ EGFR levels are dramatically elevated in numerous cancer types. Because EGFR tyrosine kinase signaling is tightly linked to cancer progression, blocking receptor activation can effectively stop tumor growth.^11,12^ Conversely, VEGFR-2, the principal angiogenic factor, binds to type III receptor tyrosine kinase and, upon interaction with VEGF produced by tumor cells, becomes significantly active on vascular endothelial cells.^13^ An incessantly active VEGFR-2 facilitates the formation of tumor vasculature, providing oxygen and nutrients to the tumor tissue, thereby expediting its growth, invasion, and metastasis.^14^ A recent study indicates that VEGFR-2 is overexpressed in lung, breast, stomach, colon, and liver malignancies.^15^

**Fig. 1:**
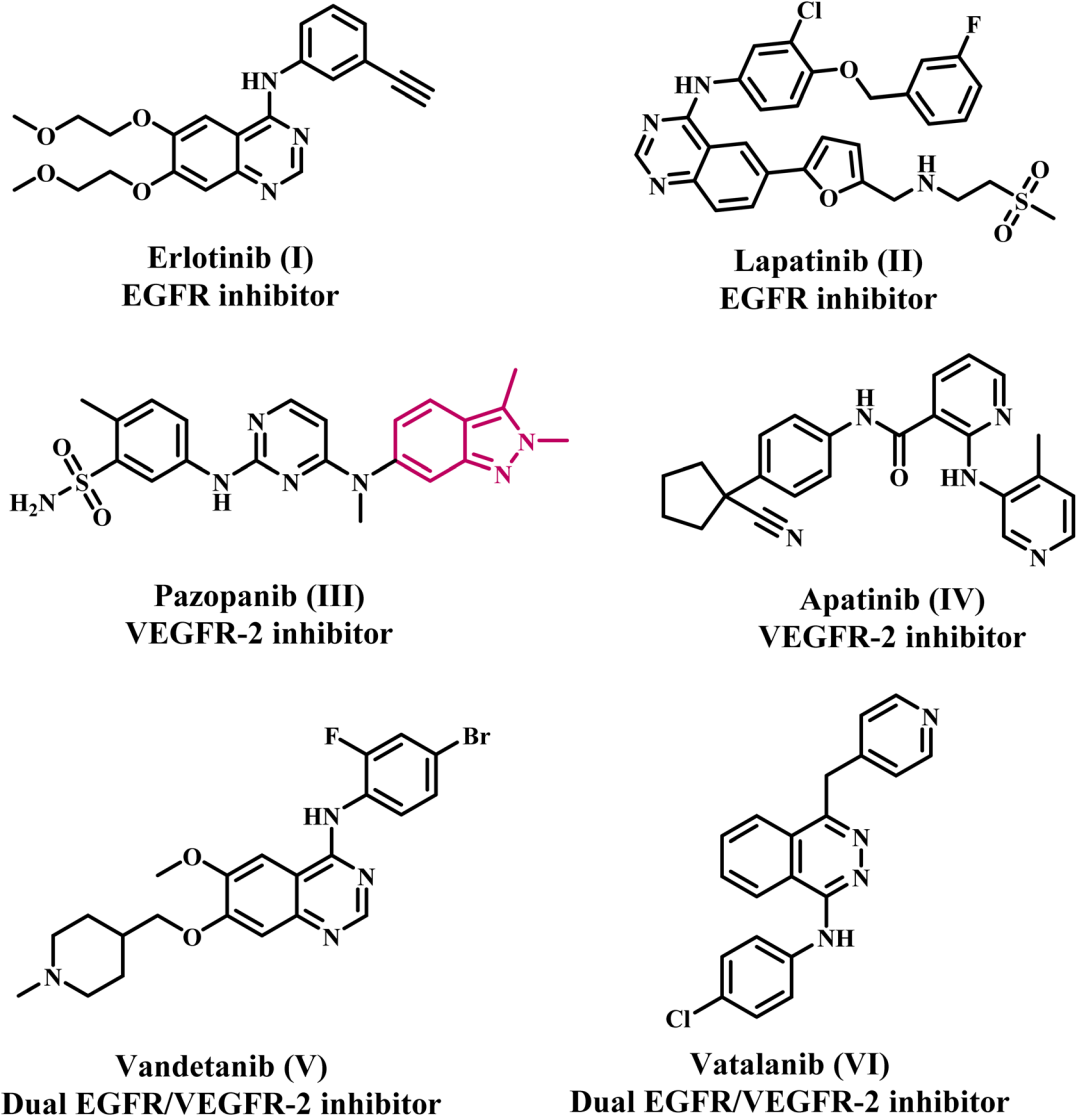
Some FDA-approved EGFR, VEGFR-2, and dual EGFR/VEGFR-2 inhibitors.

Moreover, numerous human cancers have been identified to overexpress these two kinases. A variety of therapeutically licensed anticancer agents (Fig. 1) demonstrate significant inhibitory effects on EGFR and/or VEGFR-2.^16,17^ Thus, the inhibition of both EGFR and VEGFR-2 signaling pathways is acknowledged as a viable approach for the development of novel antiproliferative agents.

The potential of heterocycles to engage in various intermolecular interactions, including π-stacking, hydrogen bonding (both as donors and acceptors), metal coordination, hydrophobic interactions, and van der Waals forces, facilitates their enhanced binding at inhibitor sites across diverse targets.^18–20^ Heteroatoms function as hydrogen bond acceptors, whilst attached N–H or O–H groups serve as donors. These interactions are essential for stabilizing ligand–protein complexes, as they can establish robust, particular interactions with amino acid residues in the binding site.^21^ Conversely, aromatic or heteroaromatic rings can participate in attractive noncovalent interactions (π–π stacking) with other aromatic residues (*e.g.*, phenylalanine, tyrosine, tryptophan) within the protein's binding pocket. The electron density of the π system, modifiable by the kind and position of heteroatoms and substituents, affects the strength and orientation (*e.g.*, face-to-face or edge-to-face) of these interactions.^22^ Additionally, The carbon-dense segments of heterocyclic structures and their associated non-polar groups engage positively with the hydrophobic areas of the protein's binding site, facilitating the displacement of water molecules and contributing substantial stability to the overall complex.^23^ The modifications of heterocyclic rings with substituents allow for extensive chemical diversity, hence enhancing their suitability as a robust scaffold for anti-cancer medication development.^24^ For instance, the pyrimidine ring is a six-membered nitrogenous heterocycle present in nucleic acids and serves as an essential framework in anti-cancer pharmaceuticals. For example, 5-fluorouracil (5-FU). The hydrogen at the 5-position of the uracil ring is substituted with a fluorine atom. This modification provides an efficient inhibitor of thymidylate synthase. The fluorine atom's diminutive size allows it to imitate hydrogen in biological activities; yet its electronic characteristics interfere with DNA synthesis and repair in cancer cells.^25^

**Fig. 2:**
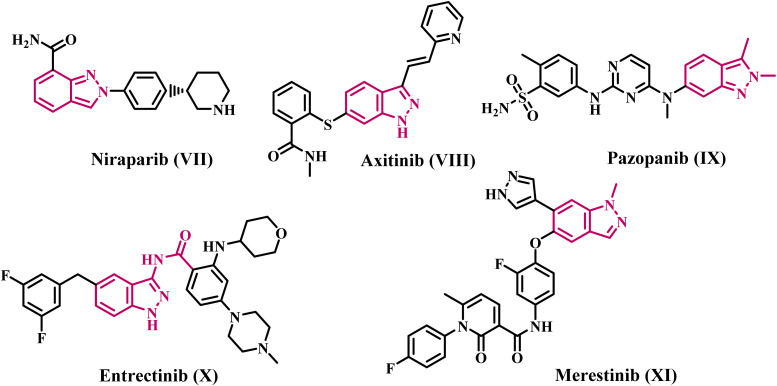
Structures of clinically approved indazole-based anticancer drugs.

Indazole is a heterocyclic molecule characterized by a bicyclic ring structure with a benzene ring and a pyrazole ring. Indazoles are bioisosteres of indoles characterized by two contiguous nitrogen atoms.^26,27^ Indazole serves as an exceptional scaffold for the development of targeted anticancer medicines that exhibit high efficacy and reduced toxicity.^28,29^ Molecules that incorporate indazole exhibit a diverse array of pharmacological activities, encompassing antitumor, antifungal, antiarrhythmic, anti-HIV, and anticancer properties.^30,31^ Over two-thirds of the novel anticancer therapeutics authorized by the Food and Drug Administration (FDA) from 2016 to 2020 incorporate an indazole moiety, leading to the advancement of anticancer agents.^32^Fig. 2 illustrates FDA-approved and commonly utilized indazole-based anticancer pharmaceuticals. All the medications depicted in Fig. 2 are tyrosine kinase inhibitors, except Niraparib (PARP inhibitor).

**Fig. 3:**
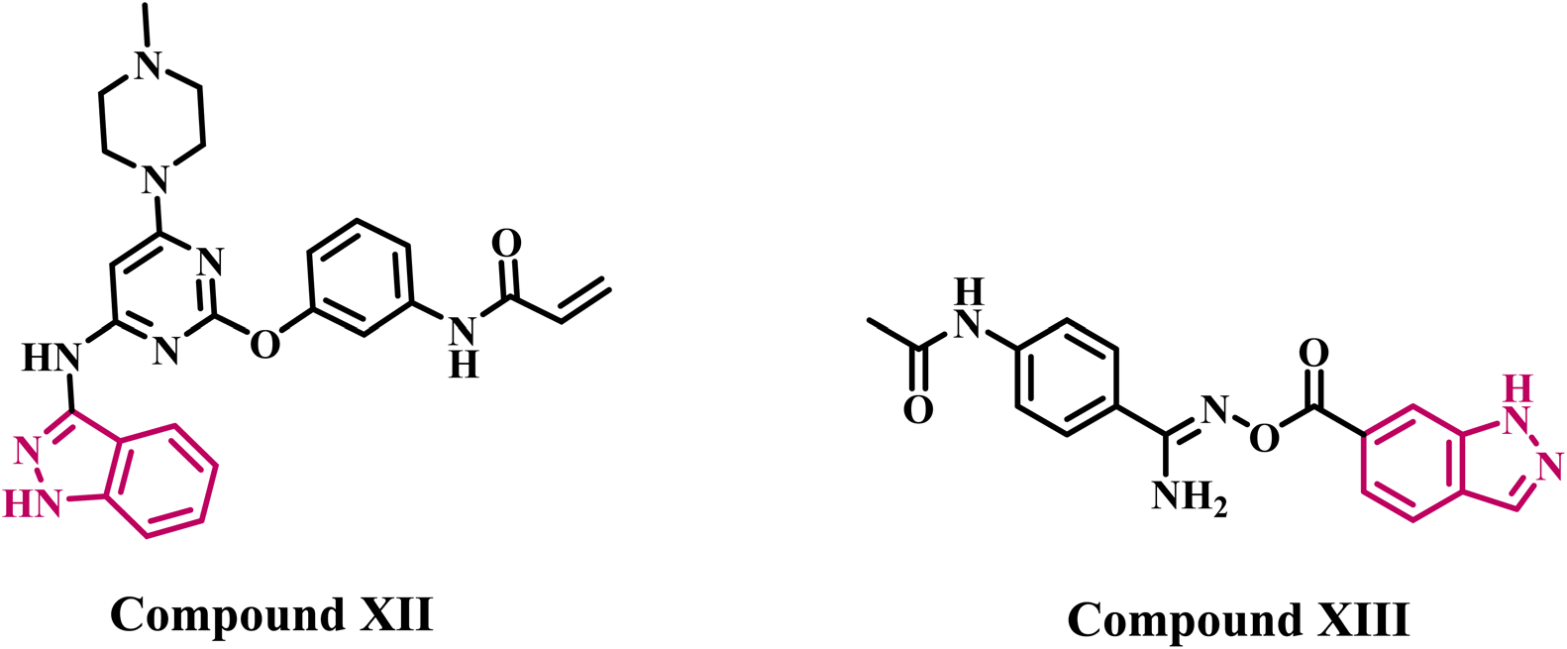
Structures of indazole-based derivatives XII and XIII as EGFR inhibitors.

Several articles have extensively discussed the development of indazole derivatives that particularly target EGFR and VEGFRs. Engel *et al.*^33^ introduced an indazole-based analog XII (Fig. 3), recognized as the most effective EGFR inhibitor within a series of indazole derivatives. Compound XII demonstrated IC_50_ values of 1.70 µM for wild-type EGFR, while exhibiting a markedly superior inhibitory impact on the drug-resistant EGFR mutant.

In a recent publication from our lab,^34^ we describe the design, synthesis, and antiproliferative efficacy of novel indazole-based derivatives as multi-target inhibitors. Compound XIII (Fig. 3) exhibited the highest efficacy as an EGFR inhibitor, demonstrating an IC_50_ value of 85 ± 5 nM, compared to the reference erlotinib, which had an IC_50_ value of 80 ± 5 nM. Compound XIII induced apoptosis by upregulating cytochrome c, activating caspases 3, 8, and 9, activating Bax, and suppressing the antiapoptotic protein Bcl-2.

**Fig. 4:**
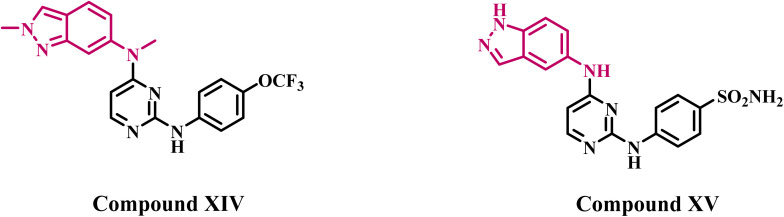
Structures of indazole-based derivatives XIV and XV as VEGFR-2 inhibitors.

Qi *et al.*^35^ investigated a series of pazopanib-based compounds that have been modified by acquiring an indazole ring. Compound XIV (Fig. 4) demonstrated the highest activity of the synthesized compounds, with an IC_50_ value of 12 nM against VEGFR-2 kinase, compared to pazopanib, which had an IC_50_ value of 30 nM. In a separate investigation,^36^ the authors identified compound XV (Fig. 4) as the most efficient derivative in a new series of indazole-based VEGFR-2 inhibitors. Compound XV demonstrated an IC_50_ value of 24.5 nM, similar to that of the reference pazopanib (IC_50_ = 25 nM).

**Fig. 5:**
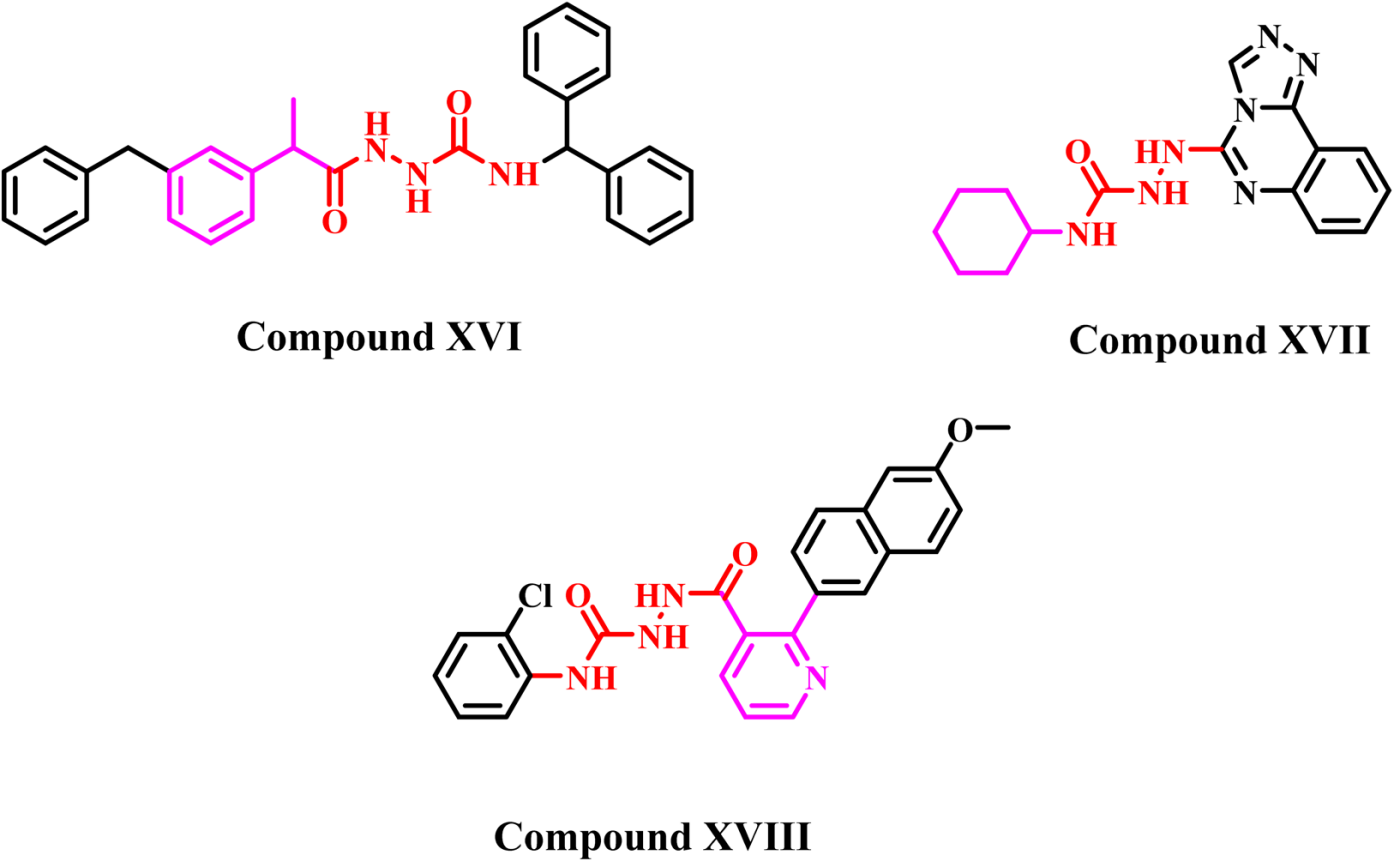
Some reported anticancer semicarbazide-containing molecules (XVI–XVIII).

Semicarbazide (NH_2_–NH–CONH_2_) is a multifunctional group that is widely identified in the chemical structures of therapeutically beneficial drugs. In addition to its diverse pharmacological effects, such as antitubercular,^37^ antioxidant,^38^ and anti-inflammatory potential,^39^ semicarbazide is widely used as a strategic pharmacophore in the development of anticancer agents,^40–42^ as shown in Fig. 5.

### 1.1. Rational design

EGFR and VEGFR-2 have been recognized as potential therapeutic targets in the battle against cancer. They are crucial elements of signaling networks that govern the angiogenesis, motility, differentiation, and proliferation of tumor cells.^43,44^ The downstream signaling pathways common to EGFR and VEGFR-2 constitute a complex network of interrelated circuits. Inhibition of EGFR can reduce VEGF production and inhibit angiogenesis. This may ultimately lead to resistance to EGFR inhibitors and an increase in VEGFR-2 expression.^45,46^ Consequently, simultaneous inhibition of both EGFR and VEGFR-2 has emerged as an effective cancer therapeutic method that operates synergistically.^47–49^

**Fig. 6:**
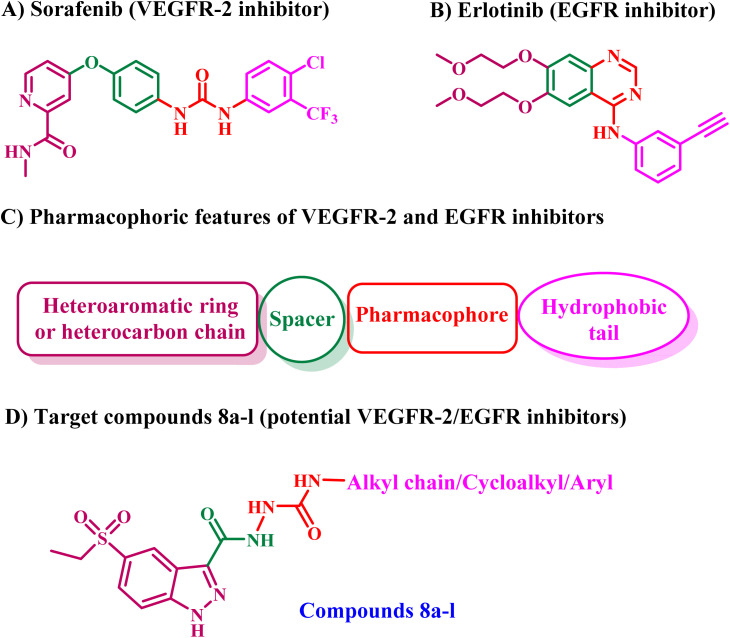
Rational design of 8a–l as dual EGFR/VEGFR-2 inhibitors.

The structure–activity relationship of FDA-approved VEGFR-2 inhibitors revealed four common characteristics: (a) a heterocyclic aromatic ring that occupies the receptor's hinge region, (b) a spacer that engages the gatekeeper region, (c) a hydrogen bonding moiety that establishes essential hydrogen bonds with the DFG amino acids, and (d) a hydrophobic tail that occupies the receptor's allosteric site,^50–52^Fig. 6A and C. Conversely, the pharmacophoric characteristics of FDA-approved EGFR inhibitors encompass a benzo-heterocyclic ring situated within the adenine binding pocket, a hydrogen bond donor or acceptor in the spacer region, a hydrophobic moiety occupying hydrophobic region I, and a hetero carbon chain serving as a hydrophobic tail interacting with hydrophobic region II (Fig. 6B and C).

Consequently, the objective of this investigation was to identify novel indazole-5-ethylsulfonyl compounds that possess the primary pharmacophore features of VEGFR-2/EGFR inhibitors (Fig. 6D).

We employed indazole-5-ethylsulfonyl to formulate novel antiproliferative drugs with potential affinity for the VEGFR and EGFR tyrosine kinases. The structures of the newly synthesized compounds (8a–l) were confirmed using ^1^H NMR, ^13^C NMR, and microanalysis. The antiproliferative effects of 8a–l were assessed *via* MTT assay on two cancer cell lines: HCT-116 (colorectal) and MCF-7 (breast). The most effective compounds from the MTT assay were further evaluated for their inhibitory efficacy against VEGFR-2 and EGFR. Also, the most effective derivatives were evaluated as apoptotic indicators for Bax, Bcl-2, p53, and caspases 3, 8, and 9. Additionally, their safety profile will be assessed in comparison to a normal human cell line, as well as their efficacy as immunomodulators. Molecular docking and dynamic simulations were conducted to examine their binding affinity at the binding sites of probable molecular targets.

## 2. Experimental

### 2.1. Chemistry

General details: Refer to Appendix A (SI).

Indazole-3-carboxylic acid 1 was obtained from Millipore Sigma and used without further purification.

#### 2.1.1. General procedure for synthesis of compounds (8a–l)

To a stirred solution of 5-(ethylsulfonyl)-1*H*-indazole-3-carbohydrazide (6) (0.37 mmol, 1 eq.) in 5 mL of dried THF, appropriate isocyanates (7a–l) (0.45 mmol, 1.2 eq.) were added, and the resultant mixture was stirred overnight. After the reaction was completed (as monitored by TLC), the reaction mixture was filtered and washed several times with THF to remove excess isocyanates. The obtained products were purified by recrystallization from ethanol.

##### 2.1.1.1. 2-(5-(Ethylsulfonyl)-1*H*-indazole-3-carbonyl)-*N*-pentylhydrazine-1-carboxamide (8a)

Yield: 0.043 g (30%), white solid, mp: 230–232 °C. ^1^H NMR (400 MHz, *δ* ppm DMSO-*d*_6_): 14.18 (s, 1H, –NH̲–N), 10.15 (s, 1H, amidic-NH̲), 8.68 (s, 1H, urea-NH̲), 7.92–7.86 (m, 3H, Ar-H), 6.43 (t, *J* = 5.8 Hz, 1H, NH̲–CH_2_), 3.32 (q, *J* = 7.3 Hz, 2H, CH̲_2_–CH_3_), 3.03 (q, *J* = 6.6 Hz, 2H, NH–CH̲_2_), 1.40 (p, *J* = 7.1 Hz, 2H, NH–CH̲_2_–CH_2_–CH_2_–CH_3_), 1.30–1.24 (m, 4H, NH–CH_2_–CH̲_2_–CH̲_2_–CH_3_), 1.11 (t, *J* = 7.3 Hz, 3H, CH_2_–CH̲_3_), 0.85 (t, *J* = 7.3 Hz, 3H, NH–CH_2_–CH_2_–CH_2_–CH̲_3_). ^13^C NMR (101 MHz, DMSO-*d*_6_): 162.1, 158.6, 142.7, 139.1, 132.8, 125.5, 123.9, 121.5, 112.8, 50.1, 31.5, 30.3, 26.4, 22.5, 14.4, 7.8. Anal. calc. (%) for C_16_H_23_N_5_O_4_S: C, 50.38; H, 6.08; N, 18.36; S, 8.40. Found: C, 50.55; H, 6.19; N, 18.30.

##### 2.1.1.2. 2-(5-(Ethylsulfonyl)-1*H*-indazole-3-carbonyl)-*N*-hexylhydrazine-1-carboxamide (8b)

Yield: 0.06 g (41%), white solid, mp: 231–233 °C. ^1^H NMR (400 MHz, *δ* ppm DMSO-*d*_6_): 14.17 (s, 1H, –NH̲–N), 10.15 (s, 1H, amidic-NH̲), 8.69 (s, 1H, urea-NH̲), 7.91–7.86 (m, 3H, Ar-H), 6.43 (t, *J* = 5.8 Hz, 1H, NH̲–CH_2_), 3.32 (q, *J* = 7.3 Hz, 2H, CH̲_2_–CH_3_), 3.03 (q, *J* = 6.3 Hz, 2H, NH–CH̲_2_), 1.39 (p, *J* = 5.8 Hz, 2H, NH–CH_2_–CH̲_2_–CH_2_), 1.30–1.23 (m, 6H, NH–CH_2_–CH_2_–(CH̲_2_)_3_–CH_3_), 1.11 (t, *J* = 7.4 Hz, 3H, CH_2_–CH̲_3_), 0.85 (t, *J* = 7.3 Hz, 3H, NH–CH_2_–CH_2_–(CH_2_)_3_–CH̲_3_). ^13^C NMR (101 MHz, DMSO-*d*_6_): 162.1, 158.6, 142.7, 139.1, 132.8, 125.5, 123.9, 121.5, 112.7, 50.1, 31.5, 30.3, 26.4, 22.6, 14.4, 7.8. Anal. calc. (%) for C_17_H_25_N_5_O_4_S: C, 51.63; H, 6.37; N, 17.71; S, 8.11. Found: C, 51.55; H, 6.43; N, 17.68.

##### 2.1.1.3. 2-(5-(Ethylsulfonyl)-1*H*-indazole-3-carbonyl)-*N*-heptylhydrazine-1-carboxamide (8c)

Yield: 0.112 g (74%), white solid, mp: 232–234 °C. ^1^H NMR (400 MHz, *δ* ppm DMSO-*d*_6_): 14.18 (s, 1H, –NH̲–N), 10.14 (s, 1H, amidic-NH̲), 8.69 (s, 1H, urea-NH̲), 7.94–7.84 (m, 3H, Ar-H), 6.42 (t, *J* = 5.7 Hz, 1H, NH̲–CH_2_), 3.32 (q, *J* = 7.3 Hz, 2H, CH̲_2_–CH_3_), 3.03 (q, *J* = 6.6 Hz, 2H, NH–CH̲_2_), 1.40 (p, *J* = 6.6 Hz, 2H, NH–CH_2_–CH̲_2_–CH_2_), 1.29–1.24 (m, 8H, NH–CH_2_–CH_2_–(CH̲_2_)_4_–CH_3_), 1.11 (t, *J* = 7.3 Hz, 3H, CH_2_–CH̲_3_), 0.85 (t, *J* = 6.7 Hz, 3H, NH–CH_2_–CH_2_–(CH_2_)_4_–CH̲_3_). ^13^C NMR (101 MHz, DMSO-*d*_6_): 161.6, 158.2, 142.3, 138.7, 132.4, 125.1, 123.4, 121.1, 112.2, 49.7, 31.3, 29.9, 28.5, 26.3, 22.1, 14.0, 7.4. Anal. calc. (%) for C_18_H_27_N_5_O_4_S: C, 52.79; H, 6.65; N, 17.10; S, 7.83. Found: C, 52.76; H, 6.61; N, 17.14.

##### 2.1.1.4. 2-(5-(Ethylsulfonyl)-1*H*-indazole-3-carbonyl)-*N*-octylhydrazine-1-carboxamide (8d)

Yield: 0.125 g (79%), white solid, mp: 245–247 °C. ^1^H NMR (400 MHz, *δ* ppm DMSO-*d*_6_): 14.18 (s, 1H, –NH–N), 10.14 (s, 1H, amidic-NH̲), 8.69 (s, 1H, urea-NH̲), 7.94–7.84 (m, 3H, Ar-H), 6.42 (t, *J* = 5.7 Hz, 1H, NH̲–CH_2_), 3.32 (q, *J* = 7.3 Hz, 2H, CH̲_2_–CH_3_), 3.03 (q, *J* = 6.6 Hz, 2H, NH–CH̲_2_), 1.40 (p, *J* = 6.6 Hz, 2H, NH–CH_2_–CH̲_2_–CH_2_), 1.29–1.24 (m, 10H, NH–CH_2_–CH_2_–(CH̲_2_)_5_–CH_3_), 1.11 (t, *J* = 7.3 Hz, 3H, CH_2_–CH̲_3_), 0.85 (t, *J* = 6.7 Hz, 3H, NH–CH_2_–CH_2_–(CH_2_)_5_–CH̲_3_). ^13^C NMR (101 MHz, DMSO-*d*_6_): 161.6, 158.2, 142.3, 138.7, 132.4, 125.1, 123.4, 121.1, 112.2, 49.7, 31.3, 29.9, 28.5, 26.3, 22.1, 14.0, 7.4. Anal. calc. (%) for C_19_H_29_N_5._O_4_S: C, 53.88; H, 6.90; N, 16.54; S, 7.57. Found: C, 53.96; H, 6.83; N, 16.47.

##### 2.1.1.5. 2-(5-(Ethylsulfonyl)-1*H*-indazole-3-carbonyl)-*N*-(cyclohexyl)hydrazine-1-carboxamide (8e)

Yield: 0.113 g (64%), white solid, mp: 250–252 °C. ^1^H NMR (400 MHz, *δ* ppm DMSO-*d*_6_): 14.16 (s, 1H, –NH̲–N), 10.12 (s, 1H, amidic-NH̲), 8.67 (s, 1H, urea-NH̲), 7.92–7.79 (m, 3H, Ar-H), 6.24 (d, *J* = 8.1 Hz, 1H, NH̲-cyclohexyl), 3.35 (q, *J* = 7.8 Hz, 2H, CH̲_2_–CH_3_), 1.85–1.45 (m, 6H, cyclohexyl), 1.34–1.13 (m, 5H, cyclohexyl), 1.10 (t, *J* = 7.3 Hz, 3H, CH_2_–CH̲_3_). ^13^C NMR (101 MHz, DMSO-*d*_6_): 161.6, 157.4, 142.3, 138.7, 132.4, 125.1, 123.4, 121.1, 112.3, 49.7, 48.2, 33.1, 25.3, 24.6, 7.4. Anal. calc. (%) for C_17_H_23_N_5_O_4_S: C, 51.89; H, 5.89; N, 17.80; S, 8.15. Found: C, 51.97; H, 5.96; N, 17.83.

##### 2.1.1.6. 2-(5-(Ethylsulfonyl)-1*H*-indazole-3-carbonyl)-*N*-(4-methylcyclohexyl)hydrazine-1-carboxamide (8f)

Yield: 0.11 g (71%), white solid, mp: 258–260 °C. ^1^H NMR (400 MHz, *δ* ppm DMSO-*d*_6_): 14.18 (s, 1H, –NH̲–N), 10.11 (s, 1H, amidic-NH̲), 8.68 (s, 1H, urea-NH̲), 7.99–7.76 (m, 3H, Ar-H), 6.20 (d, *J* = 8.0 Hz, 1H, NH̲-cyclohexyl), 3.33 (q, *J* = 7.6 Hz, 2H, CH̲_2_–CH_3_), 1.82–1.63 (m, 5H, cyclohexyl), 1.33–1.14 (m, 3H, cyclohexyl), 1.11 (t, *J* = 7.3 Hz, 3H, CH_2_–CH̲_3_), 1.01–0.91 (m, 2H, cyclohexyl), 0.85 (d, *J* = 6.5 Hz, 3H, cyclohexyl-CH̲_3_). ^13^C NMR (101 MHz, DMSO-*d*_6_): 161.6, 157.4, 142.3, 138.7, 132.4, 125.1, 123.4, 121.1, 112.3, 49.7, 48.6, 33.8, 33.0, 31.5, 22.2, 7.4. Anal. calc. (%) for C_18_H_25_N_5_O_4_S: C, 53.06; H, 6.18; N, 17.19; S, 7.87. Found: C, 53.14; H, 6.21; N, 17.12.

##### 2.1.1.7. 2-(5-(Ethylsulfonyl)-1*H*-indazole-3-carbonyl)-*N*-(adamantan-1-yl)hydrazine-1-carboxamide (8g)

Yield: 0.09 g (57%), white solid, mp: 257–259 °C. ^1^H NMR (400 MHz, *δ* ppm DMSO-*d*_6_): 14.14 (s, 1H, –NH̲–N), 10.08 (s, 1H, amidic-NH̲), 8.68 (s, 1H, urea-NH̲), 7.95–7.84 (m, 2H, Ar-H), 7.70 (s, 1H, Ar-H), 5.94 (s, 1H, NH̲-adamantyl), 3.33 (q, *J* = 7.3 Hz, 2H, CH̲_2_–CH_3_), 2.01 (t, *J* = 3.3 Hz, 3H, adamantyl), 1.91 (d, *J* = 2.9 Hz, 6H. adamantyl), 1.61 (d, *J* = 3.3 Hz, 6H, adamantyl), 1.11 (t, *J* = 7.3 Hz, 3H, CH_2_–CH̲_3_). ^13^C NMR (101 MHz, DMSO-*d*_6_): 161.9, 161.5, 157.0, 142.7, 139.6, 139.0, 132.9, 132.6, 125.5, 125.4, 124.0, 123.8, 121.4, 121.2, 112.7, 112.5, 67.5, 50.4, 50.1, 42.2, 36.6, 36.5, 29.4, 25.6, 7.8. Anal. calc. (%) for C_21_H_27_N_5_O_4_S: C, 56.61; H, 6.11; N, 15.72; S, 7.20. Found: C, 56.68; H, 6.09; N, 15.78.

##### 2.1.1.8. 2-(5-(Ethylsulfonyl)-1*H*-indazole-3-carbonyl)-*N*-(cycloheptyl)hydrazine-1-carboxamide (8h)

Yield: 0.12 g (79%), white solid, mp: 253–255 °C. ^1^H NMR (400 MHz, *δ* ppm DMSO-*d*_6_): 14.17 (s, 1H, –NH̲–N), 10.11 (s, 1H, amidic-NH̲), 8.67 (s, 1H, urea-NH̲), 7.97–7.82 (m, 2H, Ar-H), 7.78 (s, 1H, Ar-H), 6.26 (d, *J* = 8.0 Hz, 1H, NH̲-cycloheptyl), 3.67–3.58 (m, 1H, cycloheptyl), 3.31 (q, *J* = 7.3 Hz, 2H, CH̲_2_–CH_3_), 1.84–1.71 (m, 2H, cycloheptyl), 1.62–1.32 (m, 10H, cycloheptyl), 1.10 (t, *J* = 7.3 Hz, 3H, CH_2_–CH̲_3_). ^13^C NMR (101 MHz, DMSO-*d*_6_): 162.0, 157.7, 142.7, 139.0, 132.8, 125.5, 123.8, 121.5, 112.7, 50.8, 50.1, 35.3, 28.1, 24.0, 7.8. Anal. calc. (%) for C_18_H_25_N_5_O_4_S: C, 53.06; H, 6.18; N, 17.19; S, 7.87. Found: C, 53.14; H, 6.09; N, 17.31.

##### 2.1.1.9. 2-(5-(Ethylsulfonyl)-1*H*-indazole-3-carbonyl)-*N*-(4-fluorophenyl)hydrazine-1-carboxamide (8i)

Yield: 0.11 g (72%), white solid, mp: 262–264 °C. ^1^H NMR (400 MHz, *δ* ppm DMSO-*d*_6_): 14.23 (s, 1H, –NH̲–N), 10.36 (s, 1H, amidic-NH̲), 8.90 (s, 1H, urea-NH̲), 8.69 (t, *J* = 1.3 Hz, 1H, Ar-H), 8.29 (s, 1H, Ar-H), 7.95–7.85 (m, 2H, Ar-H), 7.54–7.44 (m, 2H, Ar-H), 7.15–7.04 (m, 2H, Ar-H), 3.31 (q, *J* = 7.3 Hz, 2H, CH̲_2_–CH_3_), 1.10 (t, *J* = 7.3 Hz, 3H, CH_2_–CH̲_3_). ^13^C NMR (101 MHz, DMSO-*d*_6_): 162.2, 159.0, 156.7, 156.0, 142.8, 139.0, 136.5, 136.5, 133.0, 125.6, 123.8, 121.5, 120.8, 115.7, 115.4, 112.7, 67.5, 50.1, 25.6, 7.8. Anal. calc. (%) for C_17_H_16_FN_5_O_4_S: C, 50.37; H, 3.98; N, 17.28; S, 7.91. Found: C, 50.41; H, 4.05; N, 17.33.

##### 2.1.1.10. 2-(5-(Ethylsulfonyl)-1*H*-indazole-3-carbonyl)-*N*-(benzyl)hydrazine-1-carboxamide (8j)

Yield: 0.112 g (75%), white solid, mp: 254–256 °C. ^1^H NMR (400 MHz, *δ* ppm DMSO-*d*_6_): 14.17 (s, 1H, –NH̲–N), 10.24 (s, 1H, amidic-NH̲), 8.70 (s, 1H, urea-NH̲), 8.07 (s, 1H, Ar-H), 7.90–7.86 (m, 2H, Ar-H), 7.36–7.15 (m, 5H, Ar-H), 4.26 (d, *J* = 6.0 Hz, 2H, benzyl-CH̲_2_), 3.31 (q, *J* = 7.3 Hz, 2H, CH̲_2_–CH_3_), 1.11 (t, *J* = 7.3 Hz, 3H, CH_2_–CH̲_3_). ^13^C NMR (101 MHz, DMSO-*d*_6_): 162.2, 158.8, 142.7, 141.0, 139.2, 132.9, 128.6, 127.4, 127.0, 125.5, 123.9, 121.5, 112.7, 67.5, 50.1, 43.1, 25.6, 7.8. Anal. calc. (%) for C_18_H_19_N_5_O_4_S: C, 53.86; H, 4.77; N, 17.45; S, 7.99. Found: C, 53.80; H, 4.85; N, 17.42.

##### 2.1.1.11. 2-(5-(Ethylsulfonyl)-1*H*-indazole-3-carbonyl)-*N*-(4-methoxybenzyl)hydrazine-1-carboxamide (8k)

Yield: 0.126 g (79%), white solid, mp: 244–246 °C. ^1^H NMR (400 MHz, *δ* ppm DMSO-*d*_6_): 14.19 (s, 1H, –NH̲–N), 10.24 (s, 1H, amidic-NH̲), 8.05 (s, 1H, urea-NH̲), 8.71 (s, 1H, Ar-H), 7.92–7.87 (m, 2H, Ar-H), 7.23 (d, *J* = 8.6 Hz, 2H, Ar-H), 6.88 (d, *J* = 8.7 Hz, 2H, Ar-H), 4.20 (d, *J* = 6.0 Hz, 2H, CH̲_2_-benzyl), 3.73 (s, 3H, Ar-OCH̲_3_), 3.34 (q, *J* = 7.3 Hz, 2H, CH̲_2_–CH_3_), 1.11 (t, *J* = 7.3 Hz, 3H, CH_2_–CH̲_3_). ^13^C NMR (101 MHz, DMSO-*d*_6_): 162.2, 158.7, 158.5, 142.7, 139.2, 132.9, 132.8, 128.8, 125.5, 123.9, 121.5, 114.0, 112.7, 55.5, 50.1, 42.6, 7.8. Anal. calc. (%) for C_19_H_21_N_5_O_5_S: C, 52.89; H, 4.91; N, 16.23; S, 7.43. Found: C, 53.00; H, 4.84; N, 16.31.

##### 2.1.1.12. 2-(5-(Ethylsulfonyl)-1*H*-indazole-3-carbonyl)-*N*-phenethylhydrazine-1-carboxamide (8l)

Yield: 0.1 g (65%), white solid, mp: 230–232 °C. ^1^H NMR (400 MHz, *δ* ppm DMSO-*d*_6_): 14.18 (s, 1H, –NH̲–N), 10.17 (s, 1H, amidic-NH̲), 8.70 (t, *J* = 1.3 Hz, 1H, urea-NH̲), 8.00 (s, 1H, Ar-H), 7.96–7.84 (m, 2H, Ar-H), 7.30–7.12 (m, 5H, Ar-H), 6.48 (t, *J* = 5.7 Hz, 1H, NH̲–CH_2_–CH_2_), 3.37–3.22 (m, 4H, CH̲_2_–CH_3,_ NH–CH̲_2_–CH_2_), 2.72 (t, *J* = 7.3 Hz, 2H, NH–CH_2_–CH̲_2_), 1.11 (t, *J* = 7.3 Hz, 3H, CH_2_–CH̲_3_). ^13^C NMR (101 MHz, DMSO-*d*_6_): 162.1, 158.6, 142.7, 140.1, 139.1, 132.9, 129.1, 128.8, 126.5, 125.5, 123.9, 121.5, 112.7, 67.5, 50.1, 41.5, 36.5, 25.6, 7.8. Anal. calc. (%) for C_19_H_21_N_5_O_4_S: C, 54.93; H, 5.09; N, 16.86; S, 7.72. Found: C, 54.99; H, 5.12; N, 16.79.

### 2.2. Biology

#### 2.2.1. Cell viability assay

This test assesses the safety of new compounds 8a–l on normal cell lines. The efficacy of 8a–l was tested on the normal human mammary gland epithelial cell line MCF-10A. MCF-10A cells were incubated with 50 µM of each tested compound for four days. Cell viability was assessed using the MTT assay.^11,48^ Appendix A has extra experimental details.

#### 2.2.2. Antiproliferative assay

The MTT assay^53,54^ was employed to evaluate the antiproliferative efficacy of new compounds 8a–l against two human cancer cell lines: MCF-7 (breast) and HCT-116 (colorectal carcinoma). Erlotinib was employed as the benchmark. Check Appendix A for additional experimental information.

#### 2.2.3. VEGFR-2 inhibitory assay

Using sorafenib as a reference, the most effective derivatives, 8g and 8h with potential antiproliferative capabilities, were investigated for their capacity to inhibit VEGFR-2.^47^ Refer to Appendix A for more experimental details.

#### 2.2.4. EGFR inhibitory assay

Compounds 8g and 8h were assessed for their ability to inhibit EGFR using the EGFR-TK assay.^55^ Appendix A contains more experimental details.

#### 2.2.5. Apoptotic markers assay

Compounds 8g and 8h were tested as caspases-3, 8, 9, Bax and p53 activators and as down-regulators of the anti-apoptotic protein Bcl-2 against the colorectal HCT-116 cancer cell line.^56^ Appendix A gives more details.

#### 2.2.6. IL-6 and TNF-α inhibitory assay

The effect of compounds 8g and 8h on the expression of TNF-α and IL-6 were determined using of q RT-PCR technique.^57^ See Appendix A for more details.

### 2.3. Computational studies

Molecular docking simulations for EGFR (PDB ID: 1M17) and VEGFR-2 (PDB ID: 3WZE) were validated *via* a redocking test, wherein the structures of the test proteins were held in a fixed conformation while the co-crystallized ligands (erlotinib for EGFR and sorafenib for VEGFR-2) were redocked into their corresponding crystal-binding pockets. See Appendix A for supplementary information.

## 3. Results and discussion

### 3.1. Chemistry

**Scheme 1:**
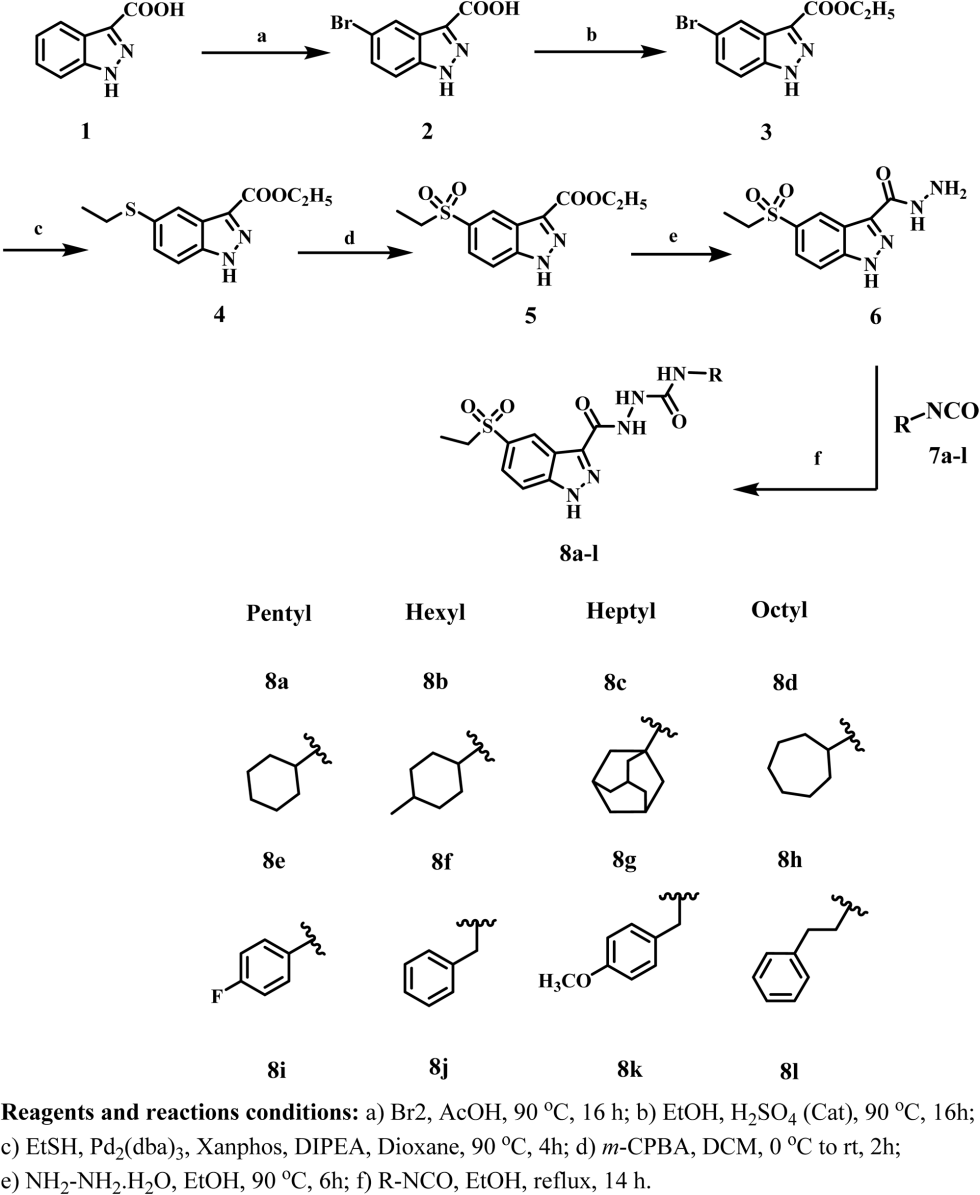
Synthetic pathway for the synthesis of new compounds 8a–l.

Compounds 8a–l were produced according to the synthesis pathway depicted in Scheme 1. The synthesis proceeded with the bromination of indazole-3-carboxylic acid 1, yielding 5-bromo-indazole-3-carboxylic acid 2 as the predominant regioisomer. Thereafter, Fischer esterification of compound 2 was performed by refluxing with anhydrous ethanol in the presence of a catalytic amount of concentrated sulfuric acid, yielding ester 3. The ^1^H NMR spectrum of compound 3 showed substantial triplet and quartet signals at *δ* 4.40 and *δ* 1.37 ppm, respectively, indicating the presence of protons in the ethyl ester group and verifying its structure (Fig. S3, SI). Subsequently, a palladium-catalyzed C–S cross-coupling reaction was conducted under Buchwald–Hartwig conditions, leading to the incorporation of a thioether functionality. Compound 4 was synthesized by reacting compound 3 with ethanethiol, employing tris(dibenzylideneacetone) dipalladium(0) [Pd_2_(dba)_3_] as the palladium catalyst, xantphos as the ligand, and DIPEA (*N*,*N*-diisopropylethylamine) as the base. Thioether 4 was further oxidized with *m*-CPBA (*m*-chloroperbenzoic acid), yielding the corresponding sulfone intermediate 5. The LC-MS analysis confirmed the synthesis of the sulfonyl derivative rather than the sulfinyl derivative, supporting the entire oxidation of the sulfur core. Furthermore, sulfone derivative 5's ^1^H NMR spectrum revealed the same number of signals as compound 4, indicating that the basic structural framework was preserved (Fig. S7, SI).

The ester 5 was then refluxed with an excess of hydrazine hydrate (79%) in absolute ethanol, producing the hydrazide derivative, compound 6. The ^1^H NMR spectrum of 6 validated the conversion, evidenced by the absence of triplet and quartet signals from the ethyl group and the identification of peaks at *δ* 5.47 and *δ* 4.84 ppm, corresponding to the –NH– and –NH_2_ protons of the hydrazide moiety (Fig. S9, SI). Finally, compounds 8a–l were prepared by refluxing hydrazide 6 with the corresponding isocyanate derivatives 7a–l in absolute ethanol for 14 h. The obtained product was washed several times with dried THF to remove excess isocyanate, followed by recrystallization from ethanol to provide pure 8a–l in good to high yields.

The structures of new compounds 8a–l were validated using ^1^H NMR, ^13^C NMR, and elemental microanalyses (Fig. S11-34, SI). The ^1^H NMR spectrum of compound 8k, as a representative example, exhibited two singlet signals at *δ* 14.19 and 10.24 ppm, attributed to the NH of the indazole and the amidic-NH, respectively. The urea proton (urea-NH̲) signal was observed at *δ* 8.05 ppm. The methylene protons (CH̲_2_-benzyl) of the benzyl moiety appeared as a doublet signal at *δ* 4.20 ppm, alongside a singlet signal from the methoxy group at *δ* 3.73 ppm. The spectrum also revealed triplet and quartet ethyl group signals at *δ* 3.34 and 1.11 ppm. The ^13^C NMR spectrum of 8k revealed characteristic signals at *δ* 162.2, 158.5, and 55.8 ppm corresponding to the carbonyl and methoxy groups.

### 3.2. Biology

#### 3.2.1. Cell viability assay

This assay evaluates the impact of novel compounds 8a–l on normal cell lines to determine their safety, an essential consideration in drug discovery. The efficacy of the examined compounds was evaluated utilizing the normal human mammary gland epithelial cell line MCF-10A. Following a four-day incubation of MCF-10A cells with 50 µM of each tested compound, cell viability was assessed using the MTT assay.^11,48^Table 1 data demonstrate that none of the evaluated compounds displayed cytotoxicity, as all compounds preserved cell viability above 90% at a dose of 50 µM.

**Table 1:** Cell viability% and IC_50_ values of compounds 8a–l^a^

Comp.	Cell viability (%)	R	Antiproliferative activity IC_50_ ± SD (nM)		
			HCT-116	MCF-7	Average IC_50_ (GI_50_)
8a	**90**	Pentyl	42 ± 4	54 ± 4	48
8b	**92**	Hexyl	33 ± 3	40 ± 3	37
8c	**91**	Heptyl	30 ± 2	34 ± 2	32
8d	**90**	Octyl	28 ± 1	31 ± 2	30
8e	**91**	Cyclohexyl	31 ± 3	39 ± 3	35
8f	**92**	4-Methylcyclohexyl	39 ± 3	50 ± 4	45
8g	**91**	Adamantyl	24 ± 1	28 ± 1	26
8h	**90**	Cycloheptyl	23 ± 1	25 ± 1	24
8i	**91**	4-Fluorophenyl	36 ± 3	44 ± 4	40
8j	**90**	Benzyl	31 ± 2	36 ± 2	34
8k	**92**	4-Methoxybenzyl	30 ± 2	32 ± 2	31
8l	**91**	Phenethyl	37 ± 3	46 ± 4	42
Erlotinib	ND	—	30 ± 2	40 ± 3	35

a ND: not determined. —: not applicable.

#### 3.2.2. Antiproliferative assay

The MTT assay^53,54^ was used to evaluate the antiproliferative properties of novel compounds 8a–l against two human cancer cell lines: MCF-7 (breast cancer) and HCT-116 (colorectal carcinoma). Erlotinib was utilized as the reference. This experiment selected two cell lines (breast and colorectal) due to evidence indicating that VEGF is significantly overexpressed in breast and colorectal tumors.^58,59^Table 1 displays the median inhibitory concentration (IC_50_). The provided values represent the means of three experiments ± standard deviation (SD).

The findings revealed that the majority of the synthesized compounds exhibited significant inhibitory activity against the tested cancer cell lines, with compounds 8a–l demonstrating greater potency against the colorectal (HCT-116) cancer cell line compared to the breast (MCF-7) cancer cell line. Compounds 8a–l showed IC_50_ values of 23 to 42 nM for the HCT-116 cell line and 25 to 54 nM for the MCF-7 cancer cell line.

Specifically, compound 8h (R = cycloheptyl) emerged as the most efficient derivative among all investigated compounds against the MCF-7 and HCT-116 cancer cell lines, with IC_50_ values of 25 and 23 nM, respectively, compared to the reference erlotinib, which displayed IC_50_ values of 40 and 30 nM for the same cell lines. Compound 8h was determined to be 1.6 times more potent than erlotinib against the breast cancer cell line MCF-7 and approximately 1.3 times more potent than erlotinib against the colorectal cancer cell line HCT-116.

The findings indicate that the nature of the substituent (R group) linked to the urea moiety is essential for its function. For instance, compound 8e (R = cyclohexyl), which shares the same backbone as compound 8h but features a cyclohexyl group in place of a cycloheptyl moiety, demonstrated IC_50_ values of 39 and 31 nM against MCF-7 and HCT-116 cancer cell lines, respectively, compared to 8h (IC_50_ values = 25 and 23 nM, respectively). Compound 8e was found to be 1.56 times less effective than 8h against the MCF-7 breast cancer cell line and approximately 1.40 times less potent than 8h against the HCT-116 colorectal cancer cell line, suggesting that the cycloheptyl moiety is more essential for antiproliferative activity than the cyclohexyl one.

Compound 8f (4-methylcyclohexyl) exhibited IC_50_ values of 50 and 39 nM against the MCF-7 and HCT-116 cancer cell lines, respectively, proving to be half as effective as 8h against the MCF-7 breast cancer cell line and 1.70-fold less potent than 8h against the HCT-116 colorectal cancer cell line. The results indicated that the structural features of the cycloalkyl moiety are crucial to the antiproliferative effects of these compounds, with activity rising in the following order: cycloheptyl > cyclohexyl > 4-methylcyclohexyl.

Compound 8g (R = adamantyl) exhibited the second highest activity, with IC_50_ values of 28 and 24 nM against the MCF-7 and HCT-116 cancer cell lines, respectively. Compound 8g exhibited efficacy comparable to that of 8h (IC_50_ values = 25 and 23 nM, respectively) against both cancer cell lines. The data indicated that both the cycloheptyl group in compound 8h and the adamantyl group in 8g are compatible with antiproliferative action.

Compound 8d (R = octyl) ranked third in activity, with IC_50_ values of 31 and 28 nM against the MCF-7 and HCT-116 cancer cell lines, respectively. The results indicate that the length of the carbon chain in the alkyl group (R) has a significant influence on the antiproliferative activity of the compounds. Compounds 8a (R = pentyl), 8b (R = hexyl), and 8c (R = heptyl) exhibited IC_50_ values of 42, 33, and 30 nM against the HCT-116 cancer cell line, respectively, compared to 28 nM for 8d against the same cell line. Additionally, their IC_50_ values against the MCF-7 breast cancer cell line were 54, 40, and 34 nM, respectively, compared to 31 nM for 8d against that cell line. The findings indicated that antiproliferative action was enhanced with the elongation of the alkyl side chain (R), following the order: octyl > heptyl > hexyl > pentyl.

Compound 8j (R = benzyl) exhibited IC_50_ values of 36 nM and 31 nM against the MCF-7 and HCT-116 cancer cell lines, respectively. Substituting the benzyl group in compound 8j with a phenethyl group, as shown in compound 8l (R = phenethyl), resulted in a significant reduction in antiproliferative activity. Compound 8l showed an IC_50_ value of 46 nM against the MCF-7 breast cancer cell line, whereas compound 8j had an IC_50_ value of 36 nM against the same cell line. Furthermore, compound 8l exhibited an IC_50_ value of 37 nM against the colorectal cancer cell line HCT-116, compared to 31 nM for compound 8j. These data indicated the substantial influence of the benzyl group compared to phenethyl on antiproliferative activity. Ultimately, compound 8k (*R* = 4-methoxybenzyl) had an IC_50_ value of 30 nM against the HCT-116 cancer cell line, comparable to that of 8j, while demonstrating an enhanced IC_50_ value against the breast cancer cell line (MCF-7). Compound 8k exhibited an IC_50_ value of 32 nM, compared to 36 nM for 8j against the MCF-7 cancer cell line.

#### 3.2.3. VEGFR-2 inhibitory assay

Using sorafenib as a reference, the most effective derivatives, 8g and 8h, with promising antiproliferative properties, were examined for their ability to inhibit VEGFR-2.^47^ Results are cited in Table 2 as IC_50_ values. All values are means of three experiments ± SD.

**Table 2:** IC_50_ values of compounds 8g and 8h against EGFR and VEGFR-2^a^

Compound	VEGFR-2 inhibition IC_50_ ± SEM (nM)	EGFR inhibition IC_50_ ± SEM (nM)
8g	16 ± 1	71 ± 5
8h	12 ± 1	68 ± 5
Sorafenib	0.17 ± 0.001	—
Erlotinib	—	80 ± 5

a ––: not determined.

The results demonstrated that compounds 8g and 8h significantly suppressed VEGFR-2, exhibiting IC_50_ values of 16 and 12 nM, respectively. Sorafenib exhibited a reduced IC_50_ value of 0.17 nM (more potent). Compounds 8g and 8h demonstrate significant antiproliferative activity and could potentially function as inhibitors of VEGFR-2.

#### 3.2.4. EGFR inhibitory assay

Compounds 8g and 8h were evaluated for their potential to inhibit EGFR using the EGFR-TK assay.^55^Table 2 presents the data, utilizing erlotinib as the reference medication. The results of this assay correspond with the findings of the antiproliferative and VEGFR-2 inhibitory assays. Compounds 8g and 8h exhibited significant EGFR inhibitory action with IC_50_ values of 71 and 68 nM, respectively, surpassing the potency of erlotinib, which had an IC_50_ value of 80 nM. These results indicate that compounds 8g and 8h exhibit promising dual inhibitory effects on EGFR and VEGFR-2, potentially serving as antiproliferative agents.

#### 3.2.5. Apoptotic markers assay

Apoptosis dysregulation is a hallmark of human cancer, resulting in uncontrolled proliferation, insufficient response to treatments, and the formation of drug-resistant cells.^60^ As a result, current anticancer therapies are recognized for their ability to induce apoptosis in cancer cells *via* both extrinsic and intrinsic routes.^56^ Therefore, compounds 8g and 8h were evaluated for their ability to induce apoptosis in HCT-116 colorectal cancer cells by examining the expression of key apoptotic markers, including Bcl-2, p53, and Bax. The findings are presented in Table 3.

**Table 3:** Apoptotic assays findings for 8g and 8h against Bax, p53, and Bcl-2

Compound no.	Bcl-2 (ng mL^−1^)	Fold reduction	Bax (pg mL^−1^)	Fold change	p53 (pg mL^−1^)	Fold change
8g	1.60 ± 0.001	3.10	490 ± 2	8.20	320 ± 2	4.90
8h	1.30 ± 0.001	3.90	510 ± 3	8.50	355 ± 2	5.50
Control	5	1	60	1	65	1

The Bcl-2 protein family, comprising pro-apoptotic proteins (Bax) and anti-apoptotic proteins (Bcl-2), primarily regulates apoptosis. Numerous studies have demonstrated a significant correlation between increased Bcl-2 levels and decreased Bax levels, which are associated with tumor cell proliferation.^61,62^ As a result, we evaluated the level of Bcl-2 and Bax proteins in HCT-116 colorectal cancer cells subjected to treatments with compounds 8g and 8h. Table 3 indicates that compound 8h produced an 8.50-fold increase in Bax levels and a 3.90-fold decrease in Bcl-2 levels relative to control, untreated cells. Moreover, compound 8g demonstrated an 8.20-fold elevation in Bax levels and a three-fold reduction in Bcl-2 levels. These observations suggest that apoptosis may play a role in the antiproliferative activities of the investigated compounds.

The ability of p53 overexpression to trigger apoptosis may clarify the common inactivation of p53 enzymes by cancer cells during transformation.^63^ The p53 levels in cancer cells treated with compounds 8g and 8h exhibited a substantial increase, surpassing those of the untreated control cells by at least 5-fold. This observation indicates that higher levels of the p53 protein govern the apoptotic process in these new compounds.

Additionally, activating caspases is essential for both initiating and terminating the apoptotic process.^64^ Caspase-3 is an essential enzyme that cleaves several proteins within cells, leading to apoptotic cell death.^65^ The effects of compounds 8g and 8h on caspase-3 were evaluated using the colorectal (HCT-116) cancer cell line and compared to staurosporine as a reference drug (Table 4). The results demonstrated that 8h was the most effective derivative, showing a notable overexpression of caspase-3 protein levels (510 ± 4 pg mL^−1^), compared to the reference staurosporine (465 ± 4 pg mL^−1^). Compound 8h demonstrated a 7.90-fold increase in active caspase-3 levels relative to control HCT-116 cells and elicited caspase-3 levels akin to those generated by staurosporine, the reference drug. Compound 8g exhibited a 7.50-fold elevation in active caspase-3 levels (490 ± 3 pg mL^−1^) relative to the control untreated colorectal (HCT-116) cells, as indicated in Table 4.

**Table 4:** Caspases 3, 8, and 9 assays of compounds 8g and 8h

Compd no.	Caspase-3		Caspase-8		Caspase-9	
	Conc. (pg ml^−1^)	Fold change	Conc. (ng ml^−1^)	Fold change	Conc. (ng ml^−1^)	Fold change
8g	490 ± 5	7.50	1.80 ± 0.10	18.00	21 ± 3	21
8h	510 ± 5	7.90	2.15 ± 0.20	21.50	23 ± 1	23
Staurosporine	465 ± 4	7.00	1.85 ± 0.10	18.50	20 ± 1	20
Control	65	1.0	0.10	1	1	1

To clarify the apoptotic mechanisms of compounds 8g and 8h, whether through the intrinsic or extrinsic pathway, their effects on caspase-8 and caspase-9 were assessed. The findings demonstrated that compound 8h enhances the levels of caspase-8 and caspase-9 by 21- and 23-fold, respectively, while compound 8g raises the levels of caspase-8 and caspase-9 by 18- and 21-fold, respectively, relative to the control HCT-116 cancer cells. This indicates the activation of both intrinsic and extrinsic pathways, with a more significant impact on the intrinsic pathway, as demonstrated by the increased levels of caspase-9 (Table 4).

#### 3.2.6. *In vitro* cytotoxicity against normal human cells

Consequently, it was crucial to assess the safety profiles of the most potent compounds, 8g and 8h, on the normal human diploid cell line (WI-38) using the MTT assay^66^ to determine the selectivity of the target compounds for cancer cells compared to normal cells. The evaluated compounds 8g and 8h demonstrated IC_50_ values over 150 nM. The results indicated a favorable safety margin for the evaluated compounds about normal cells, Table 5.

**Table 5:** IC50 values of compounds 8g and 8h against normal cell line (WI-38)

Compound	Cytotoxicity (WI-38) IC_50_ (nM)	Selectivity index (SI)	
		HCT-116	MCF-7
8g	>150	>6.0	>5.0
8h	>150	>6.5	>6.0

#### 3.2.7. Effects on the levels of TNF-α and IL-6 (immunomodulatory) proteins

Cytokines play a crucial role in the progression and spread of cancer. Comprehensive research is being conducted on anti-cytokine medicines, which may lead to novel treatments for symptoms that are currently difficult to manage.^67^ Interleukin-6 (IL-6) and tumor necrosis factor-alpha (TNF-α) are multifunctional cytokines associated with tumor proliferation and metastasis.^68,69^ TNF-α has been linked to cancer progression and spread in both human and experimental models.^70^ As a result, anticancer drugs that inhibit both TNF-α and IL-6 are beneficial for pharmaceutical development. The effects of the most active compounds 8g and 8h on the levels of immunomodulatory proteins (TNF-α and IL-6) were evaluated by qRT-PCR.^57^ HCT-116 cells were treated with compounds 8g and 8h for 24 hours at doses of 24 nM and 23 nM (IC_50_ against HCT-116), respectively. The reference molecule was dexamethasone, a drug that uniformly regulates the immune system. Compounds 8g and 8h exhibited a notable decrease in TNF-α levels, with inhibition rates of nearly 80%, as indicated in Table 6, comparable to the value of 83% for dexamethasone. Compound 8h showed significant suppressive effects on IL-6 (91%) compared to dexamethasone (93%), whereas compound 8g revealed an 86% suppression of IL-6.

**Table 6:** % Inhibition of compounds 8g and 8h against TNF-α and IL-6

Compound	TNF-α (% inhibition)	IL-6 (% inhibition)
8g	79	86
8h	82	91
Dexamethasone	83	93

### 3.3. Computational approaches

#### 3.3.1. Molecular mechanics-based simulations

Molecular mechanics (MM) methods, grounded in classical Newtonian physics, provide a foundational framework for exploring molecular conformation, energetic profiles, and non-covalent interactions.^71^ In this study, MM principles were integrated into molecular docking and dynamics simulations to elucidate the binding landscape of the synthesized urea derivatives (8e and 8h) within kinase targets, such as EGFR and VEGFR-2. By simulating energy surfaces and conformational preferences, MM-based calculations offered insight into how subtle variations in the side chains (*e.g.*, cycloheptyl *vs.* cyclohexyl) influence binding stability and receptor engagement. Furthermore, MM-derived scoring functions were instrumental in predicting ligand-receptor affinities and identifying critical interactions that govern inhibitory activity.^72^ When combined with dynamic trajectory analyses, this approach enabled an in-depth examination of compound flexibility, accommodation within active sites, and the temporal evolution of key binding interactions.^73^ These computational findings complemented the experimental antiproliferative results, offering a mechanistic rationale for the observed structure–activity relationships and guiding future optimization of kinase-inhibiting scaffolds.^74^

#### 3.3.2. Molecular docking simulations

To explore the binding preferences and molecular recognition patterns of selected indazole-based analogs, compounds 8e and 8h were subjected to docking analyses against EGFR (PDB ID: 1M17) and VEGFR-2 (PDB ID: 3WZE).^75,76^ The FDA-approved kinase inhibitors erlotinib and sorafenib were employed as benchmarks for EGFR and VEGFR-2, respectively. Crystallographic coordinates were retrieved from the Protein Data Bank, and protein preprocessing was performed using the CDOCKER module in Discovery Studio 2016, which leverages the CHARMm force field.

**Fig. 7:**
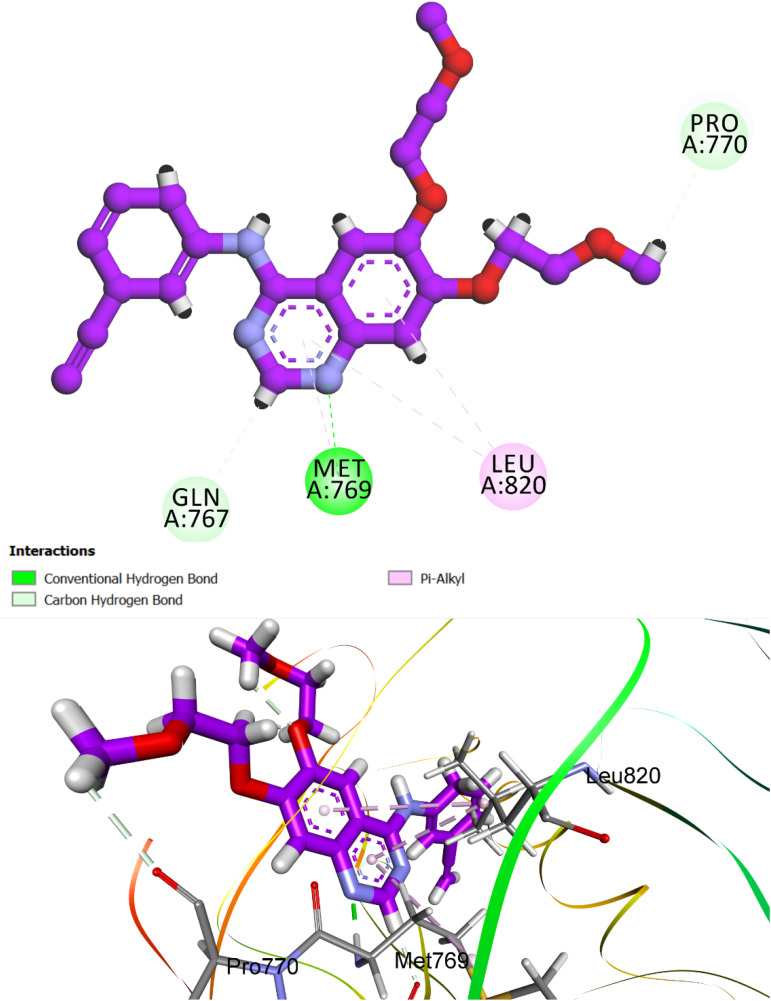
Superimposed validation pose of the reference ligand erlotinib re-docked into the EGFR active site (PDB ID: 1M17). The pose demonstrates close agreement with the crystallographic orientation (RMSD = 1.13 Å), confirming the robustness and accuracy of the docking protocol. Key interactions, including hydrogen bonding with hinge residue Met769, are preserved, validating the docking methodology for subsequent ligand-binding predictions.

Before docking, protein structures were refined by removing all heteroatoms and solvent molecules beyond 5 Å from the binding site. Hydrogen atoms were added, and protonation states of titratable residues were optimized at physiological pH (7.4) using built-in pKa predictors. Special attention was given to histidine tautomers, ensuring accurate hydrogen bonding capabilities within the binding cleft. Energy minimization of the receptor structure was performed with a convergence threshold of 0.01 kcal mol^−1^ Å^−1^ RMS gradient to eliminate steric clashes while preserving backbone integrity. A rigid receptor–flexible ligand protocol was implemented, allowing full torsional flexibility for ligands while keeping the receptor static. Ligand structures were processed using Discovery Studio's Prepare Ligands workflow, which included 3D geometry optimization and protonation state assignment.^77^ The docking grid was centered on the coordinates of the co-crystallized ligands, targeting conserved active-site residues essential for kinase–ligand interaction fidelity.^78,79^ No blind docking was applied. For each ligand, ten poses were generated. The top-ranked pose, selected based on the CDOCKER interaction energy (incorporating both van der Waals and electrostatic contributions), was analyzed for key intermolecular interactions, including hydrogen bonding, pi–pi stacking, and hydrophobic contacts, using integrated visualization tools. To confirm the reliability of the docking protocol, self-docking validation was performed by reintroducing the native ligand into its crystallographic binding site. The resulting RMSD of 1.13 Å and a re-docking score of −7.22 kcal mol^−1^ confirmed strong concordance with experimental geometry and validated the predictive capability of the docking workflow. Notably, the canonical hinge-region hydrogen bond between the pyrimidine nitrogen of the ligands and Met769 in EGFR was preserved, reinforcing its critical role in anchoring the compounds within the ATP-binding domain (Fig. 7).

**Fig. 8:**
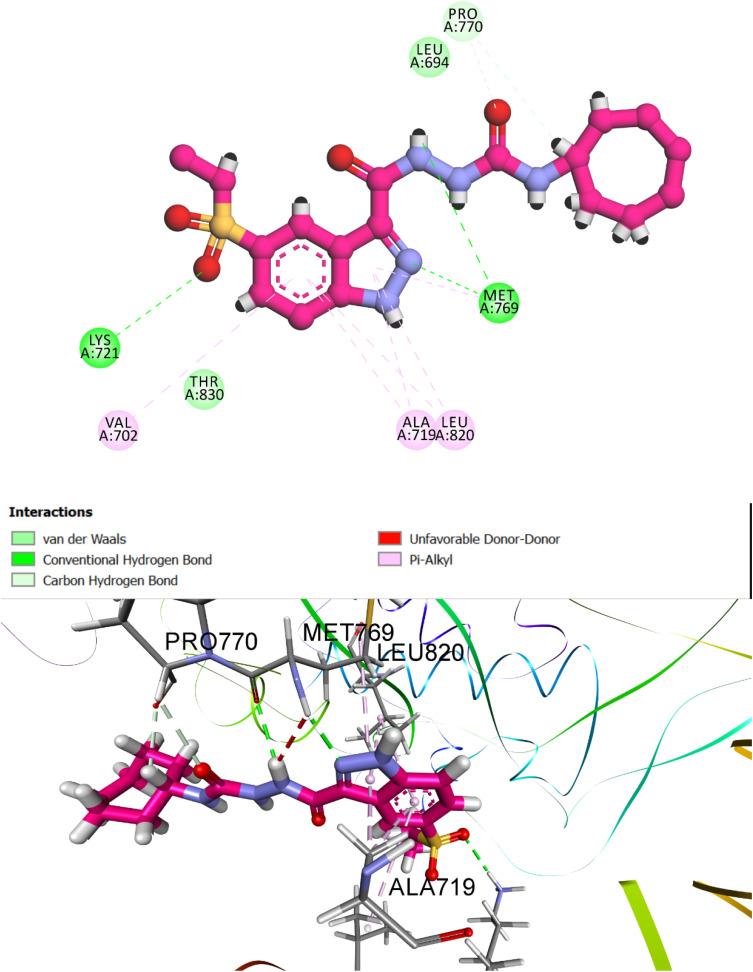
Predicted binding pose of compound 8h within the EGFR active site (PDB ID: 1M17). The indazole moiety forms hydrogen bonds with Met769 (hinge region), while the urea linker bridges interactions with Met769 and Pro770. The cycloheptyl tail occupies a peripheral hydrophobic region, contributing to anchoring and spatial orientation. Interaction types include hydrogen bonding (green), van der Waals (light green), and π-alkyl interactions (purple), with crucial contacts highlighted.

Docking results were evaluated using a combination of quantitative and qualitative metrics. The primary selection criterion was the CDOCKER interaction energy score, incorporating van der Waals and electrostatic contributions. Among all synthesized derivatives, compound 8h exhibited the strongest docking affinity, achieving an *S*-score of −7.79 kcal mol^−1^ and an RMSD of 1.63 Å, consistent with its superior experimental EGFR inhibitory activity (IC_50_ = 68 nM) compared to reference drug erlotinib (IC_50_ = 80 nM). Visual inspection of the top-scoring pose confirmed an optimal fit within the EGFR active site, with key pharmacophoric elements of 8h adopting conformations conducive to strong molecular recognition (Fig. 8). Compound 8h features a tripartite pharmacophore, comprising an indazole core, a urea linker, and a hydrophobic cycloheptyl tail. This structural triad collectively orchestrates high-affinity binding within the ATP-binding domain of EGFR. Notably, the bulky cycloheptyl moiety, while sterically hindered from entering the deep binding pocket, plays a critical allosteric role. Its size forces an outward anchoring conformation that facilitates deeper insertion of the indazole scaffold into the hinge region, thereby enhancing ligand orientation and receptor engagement.

Specifically, the indazole ring is deeply buried within the active site, forming conventional hydrogen bonds with Met769 (hinge region), a hallmark interaction essential for ATP-competitive inhibition. Additional π-alkyl contacts were observed with residues Ala719, Leu820, and Val702, further stabilizing the complex. The urea linker, central to molecular flexibility and electron delocalization, engages in dual hydrogen bonding with Met769 and Pro770, contributing both rigidity and directional orientation. Meanwhile, the cycloheptyl tail, though solvent-exposed, enhances binding enthalpy without disrupting the core interactions. Overall, compound 8h demonstrates a well-coordinated interaction profile, with each pharmacophoric segment participating in a distinct yet synergistic manner. The docking pose not only rationalizes the experimentally observed potency but also highlights the importance of steric modulation at the hydrophobic terminus to achieve optimal receptor accommodation and inhibitory performance.

**Fig. 9:**
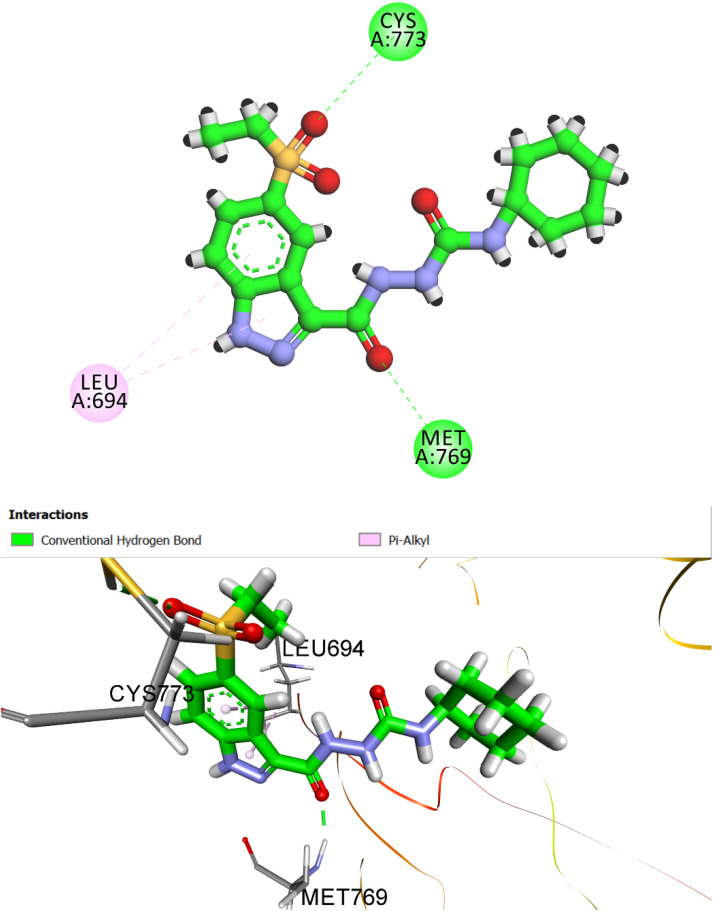
Predicted docking pose of compound 8e in the EGFR active site (PDB ID: 1M17). The urea linker forms hydrogen bonds with Met769, while the cyclohexyl moiety penetrates the binding pocket. This inward positioning of the cyclohexyl group displaces the indazole core from the hinge region, preventing critical interactions and thereby compromising the overall binding orientation and affinity.

To complement the experimental findings, compound 8e was evaluated for its binding conformation and receptor interaction landscape within the EGFR active site (PDB ID: 1M17). The docking assessment yielded an *S*-score of −6.34 kcal mol^−1^ and an RMSD of 1.78 Å, indicating weak binding affinity. The conformational pose and interaction map are depicted in Fig. 9. Compound 8e preserves the same core pharmacophore as 8h, composed of an indazole scaffold, a urea linker, and a hydrophobic tail (cyclohexyl). However, key deviations in binding behavior emerge due to the reduced steric bulk of the cyclohexyl group compared to cycloheptyl. The smaller cyclohexyl moiety readily enters the binding pocket, occupying spatial regions typically reserved for the more interactive indazole system. As a result, the indazole ring is displaced away from the hinge region, impairing its ability to engage in pivotal hydrogen bonding interactions, particularly with Met769, a residue central to ATP-competitive inhibition. Despite this suboptimal orientation, compound 8e retains some stabilizing interactions.

The urea linker maintains directional hydrogen bonds with Met769, offering partial anchoring within the catalytic cleft. Additionally, π-alkyl interactions are observed between the aromatic ring of indazole and Leu694, though these are less pronounced compared to 8h. The cyclohexyl tail, now positioned deeper in the hydrophobic region, fails to reinforce a productive ligand conformation due to its lack of steric steering capability.

In summary, the diminished binding affinity of compound 8e can be attributed to pharmacophoric misalignment, where the cyclohexyl group intrudes into the core binding cavity, excluding the indazole system from forming critical hinge-region contacts. This reversal in binding orientation highlights the importance of tailored hydrophobic bulk in modulating conformational dynamics and maximizing kinase inhibition potential.

**Fig. 10:**
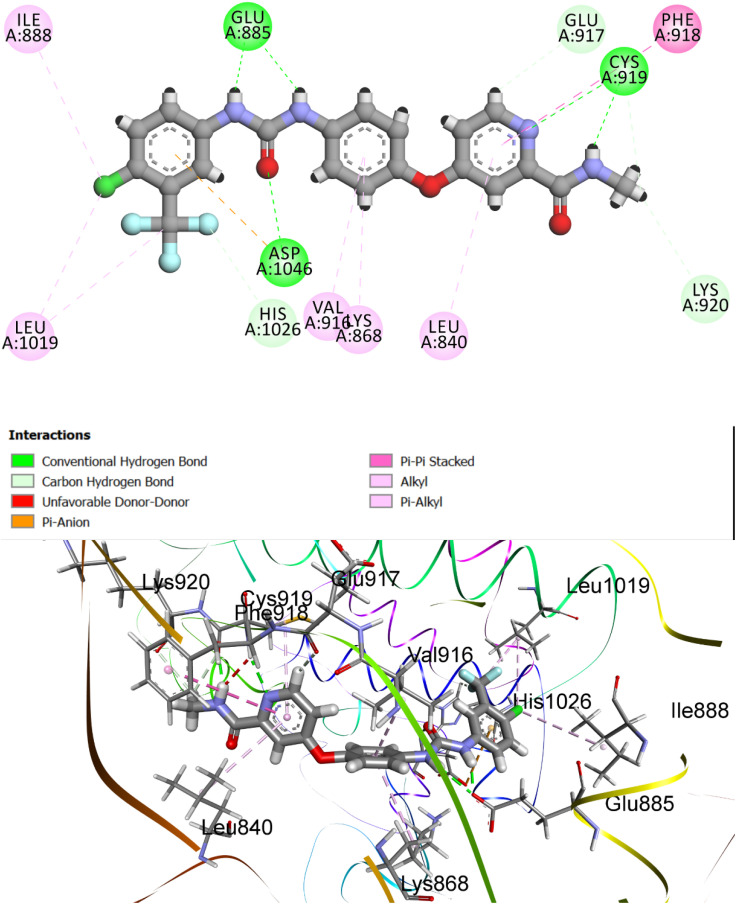
Predicted binding pose and 2D interaction map of the reference ligand sorafenib within the VEGFR-2 active site (PDB ID: 3WZE). The docking pose demonstrates excellent agreement with experimental geometry (RMSD = 1.47 Å) and a strong binding affinity (*S*-score = −8.56 kcal mol^−1^). Key stabilizing interactions include hydrogen bonds with Glu885, Cys919, and Asp1046, as well as hydrophobic contacts involving Val916, Leu840, Lys868, and Leu889. A significant π–π–π-anion interaction with Asp1046 further reinforces high-affinity binding.

To deepen our understanding of ligand–receptor recognition, molecular docking simulations were also conducted against VEGFR-2 (PDB ID: 3WZE). The reference ligand sorafenib, a clinically approved VEGFR-2 inhibitor, was employed to validate the docking protocol and benchmark the performance of synthesized analogs. Sorafenib yielded a CDOCKER interaction energy (*S*-score) of −8.56 kcal mol^−1^ with an RMSD of 1.47 Å, confirming the reliability and accuracy of the computational setup in reproducing experimentally validated binding poses (Fig. 10).

Detailed interaction analysis revealed that sorafenib established a robust network of conventional hydrogen bonds involving the key residues Glu885, Cys919, and Asp1046 within the ATP-binding cleft, which is critical for effective kinase inhibition. In addition, the ligand formed multiple hydrophobic contacts, including π–π-alkyl and alkyl interactions with Val916, Leu840, Lys868, and Leu889, thereby stabilizing its orientation within the hydrophobic cavity. Notably, a pi–anion interaction was observed with Asp1046, contributing to enhanced electrostatic complementarity.

**Fig. 11:**
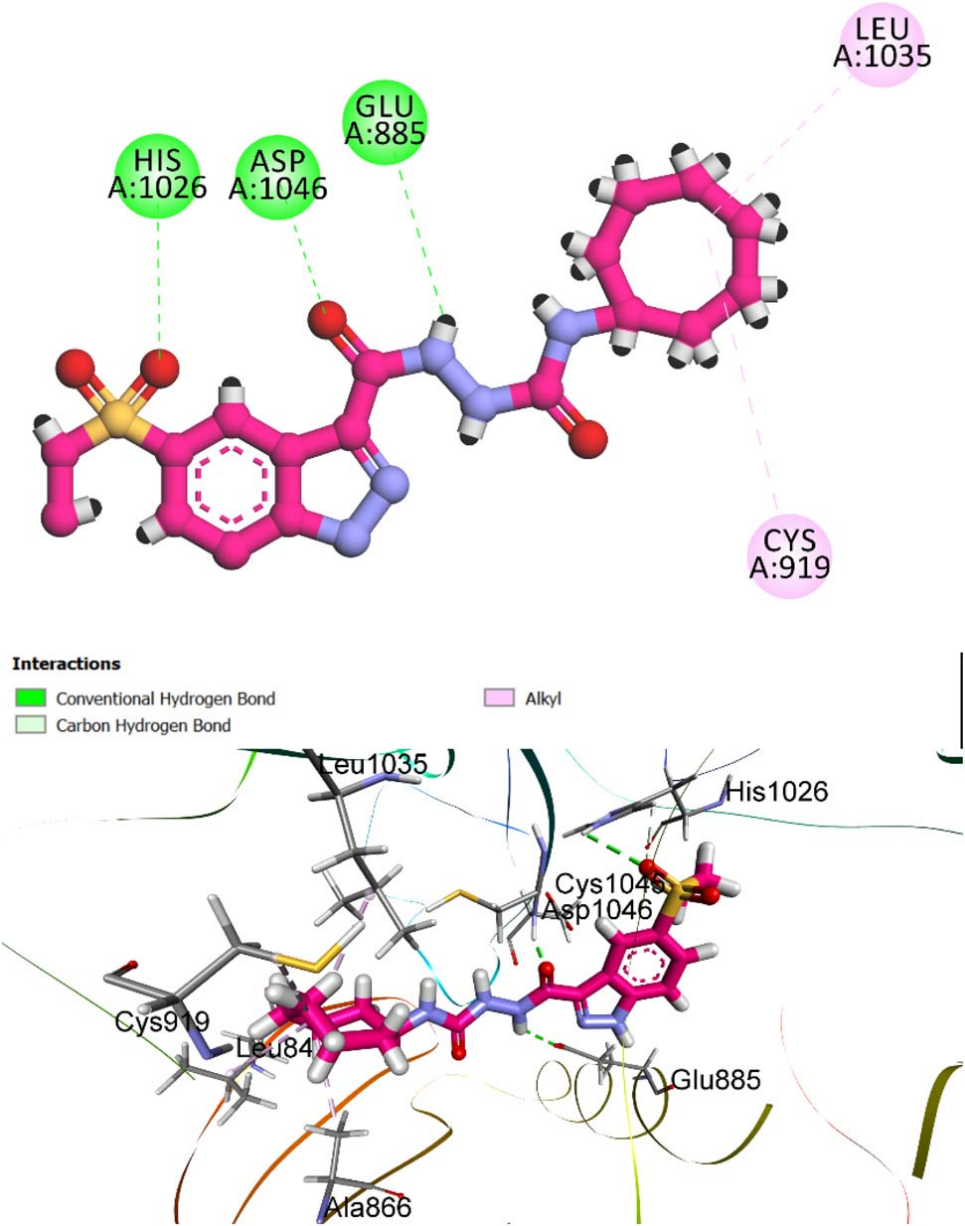
Predicted binding pose and 2D interaction diagram of compound 8h within the VEGFR-2 active site (PDB ID: 3WZE). The indazole moiety and urea linker form conventional hydrogen bonds with Glu885, Asp1046, and His1026, mimicking the interaction pattern of sorafenib. While the cycloheptyl tail engages in alkyl interactions with Cys919 and Leu1035, enhancing hydrophobic occupancy and anchoring the ligand in the receptor cleft.

Compound 8h, the most potent derivative in the series, exhibited remarkable inhibitory activity against VEGFR-2 (IC_50_ = 12 nM), a result that was strongly supported by its docking performance. The simulated binding pose returned an *S*-score of −7.28 kcal mol^−1^ and an RMSD of 1.72 Å, indicating a highly favorable and accurate fit within the VEGFR-2 binding cavity (Fig. 11). These values closely approximate those of the reference inhibitor sorafenib, reinforcing the potential of 8h as a VEGFR-2-targeted anticancer agent.

In the docked conformation, each structural moiety plays a distinct and complementary role in stabilizing the ligand–receptor complex. The sulphonyl group of indazole moiety is embedded within the ATP-binding cleft, forming hydrogen bond with His1026 residue known to be critical for kinase inhibition. The urea linker bridges the aromatic core and the hydrophobic tail, contributing to conformational rigidity and hydrogen bond alignment. It anchors the ligand *via* hydrogen bonding with Glu885, and Asp1046 residues, reinforcing the spatial orientation necessary for optimal pocket occupancy.

These interactions mirror those formed by sorafenib, suggesting a conserved binding mode and contributing significantly to binding enthalpy. The cycloheptyl tail, while larger than typical aliphatic groups, plays a unique role. It extends into a hydrophobic sub-pocket, where it engages in alkyl interactions with Cys919 and Leu1035, residues that lie along the outer rim of the binding cleft. Unlike smaller rings, the bulk of the cycloheptyl group does not forcefully invade the deep pocket but instead allows the indazole scaffold to insert properly enhancing productive interactions at the hinge region. Together, these interactions demonstrate that the steric balance and spatial distribution of functional groups in compound 8h enable a high-affinity and pharmacologically meaningful interaction profile, correlating with its potent inhibitory activity *in vitro*.

In summary, compound 8h demonstrated strong docking affinity toward both EGFR (*S*-score: −7.79 kcal mol^−1^) and VEGFR-2 (*S*-score: −7.28 kcal mol^−1^), aligning with its potent experimental activity. Its indazole moiety formed key hydrogen bonds with hinge-region residues, the urea linker provided structural stability, and the bulky cycloheptyl tail enhanced orientation by preventing deep pocket intrusion, altogether supporting its role as a promising dual kinase inhibitor.

#### 3.3.3. Molecular dynamics (MD) simulations of 8h and erlotinib with EGFR

To further elucidate the binding stability and dynamic interaction profile of 8h with EGFR, molecular dynamics (MD) simulations were performed for 80 ns using GROMACS 2023, with erlotinib serving as the reference ligand.^80^ Protein–ligand complexes were initially prepared in UCSF Chimera, where hydrogen atoms were added to ensure correct geometry and bonding.

The CHARMM36 force field was applied to the protein, and ligand topologies were generated using the CHARMM General Force Field (CGenFF) *via* the ParamChem web server.^81,82^ All assigned parameters showed penalty scores below 10, confirming high reliability and eliminating the need for manual reparameterization. The resulting complexes were solvated in a periodic cubic box filled with TIP3P water molecules, maintaining a 1 nm buffer on all sides.^83^ To replicate physiological conditions, Na^+^ and Cl^−^ ions were introduced at a concentration of 150 mM to neutralize the system. Energy minimization was conducted using the steepest descent algorithm to remove steric clashes, followed by 100 ps of equilibration under both NVT and NPT ensembles.^84^ Temperature and pressure were maintained at 300 K and 1.0 bar using the V-rescale thermostat and Parrinello–Rahman barostat, respectively.^85^

During equilibration, heavy atoms of the protein–ligand complex were position-restrained. An 80 ns production run followed, using a 2 fs integration step and saving trajectory snapshots every 10 ps. Periodic boundary conditions were applied in all directions, with bond constraints involving hydrogen atoms enforced using the LINCS algorithm.^86^ Long-range electrostatic interactions were treated using the Particle Mesh Ewald (PME) method with a cutoff of 10 Å.^87^ System stability and interaction behavior were assessed through several post-simulation metrics, including RMSD for global conformational stability, RMSF to evaluate residue-level fluctuations, and the radius of gyration (*R*_g_) to measure protein compactness. Additionally, hydrogen bond analysis was conducted to monitor the number and persistence of intermolecular hydrogen bonds throughout the simulation, and the potential energy profile was examined to confirm the attainment of thermodynamic equilibrium.

**Fig. 12:**
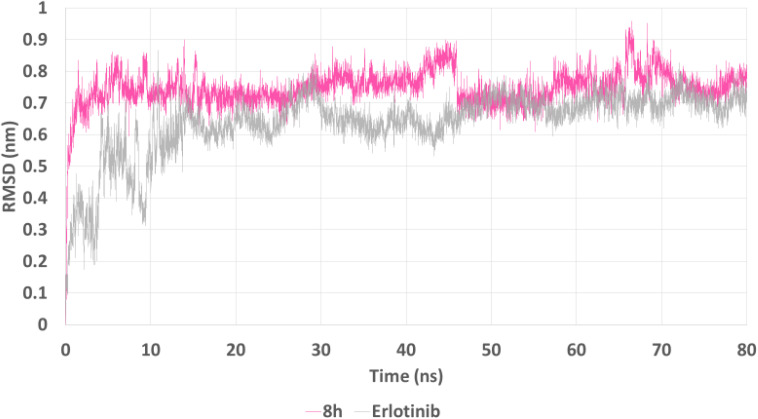
RMSD plot of the EGFR–ligand complexes over 80 ns. Compound 8h (pink) and erlotinib (gray) show rapid stabilization within the first 10 ns. 8h maintained slightly higher RMSD values, indicating moderate conformational flexibility while preserving stable complex formation throughout the simulation.

Root Mean Square Deviation (RMSD) plots (Fig. 12) showed that both ligands reached equilibrium within the first 10 ns, indicating stable complex formation. However, 8h maintained a slightly higher RMSD (∼0.75–0.85 nm) compared to erlotinib (∼0.65–0.75 nm), suggesting more pronounced conformational adaptability. Despite this, 8h displayed consistent fluctuations without significant deviation, indicating a dynamically stable interaction with EGFR over the course of the simulation.

**Fig. 13:**
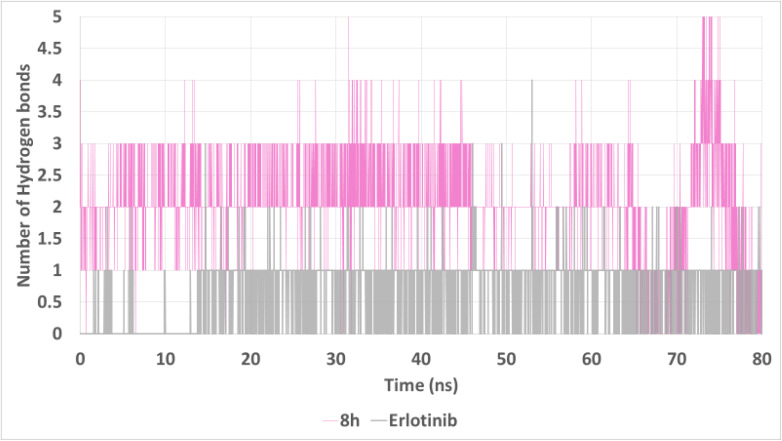
Hydrogen bond analysis between EGFR and the ligands over 80 ns. Compound 8h consistently formed 2–4 hydrogen bonds, exceeding those of erlotinib, which primarily maintained 1–2. This suggests enhanced and more persistent intermolecular interactions for 8h within the EGFR binding site.

Hydrogen bond analysis (Fig. 13) revealed a notable advantage for 8h, which formed 2–4 stable hydrogen bonds throughout the simulation, in contrast to erlotinib, which typically maintained only 1–2 hydrogen bonds. The sustained H-bond formation by 8h reflects strong and persistent interactions with critical residues in the binding pocket, further reinforcing its binding stability.

**Fig. 14:**
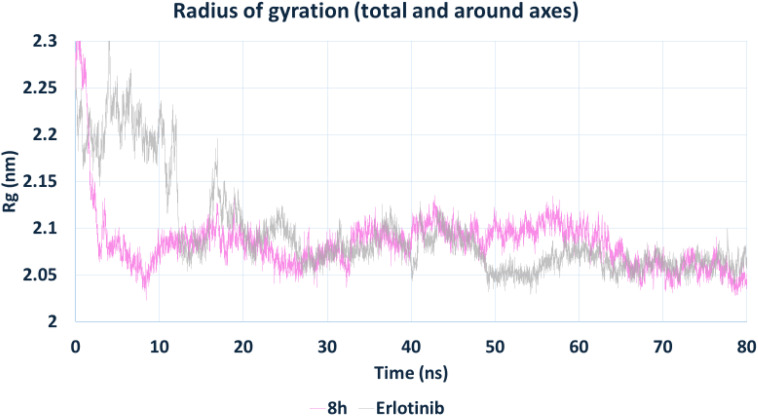
Radius of gyration (*R*_g_) of EGFR in complex with compound 8h and erlotinib. Both complexes exhibited stable *R*_g_ values throughout the simulation, with 8h promoting a slightly more compact protein conformation (∼2.05–2.10 nm), indicative of tighter structural packing.

The radius of gyration (*R*_g_) (Fig. 14), a measure of protein compactness, indicated that the EGFR–8h complex maintained a slightly more compact structure (∼2.05–2.10 nm) than the EGFR–erlotinib complex. This suggests that 8h may induce subtle conformational tightening of the receptor, potentially enhancing binding affinity by reducing structural entropy.

**Fig. 15:**
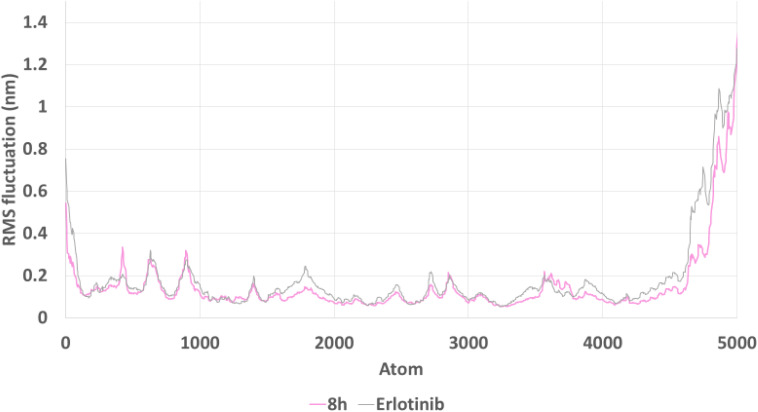
Root Mean Square Fluctuation (RMSF) of EGFR residues in complex with compound 8h and erlotinib. Residue-level fluctuations were generally low across both systems, 8h induced slightly reduced flexibility in active-site regions, reflecting localized stabilization of the binding environment.

RMSF analysis (Fig. 15) assessed residue-level flexibility. Both complexes displayed low fluctuations across most residues, indicating overall protein rigidity. However, 8h induced slightly lower RMSF values, particularly in the active-site region, suggesting a stabilizing effect on local residues. This reduction in residue flexibility may contribute to improved ligand retention within the binding site.

**Fig. 16:**
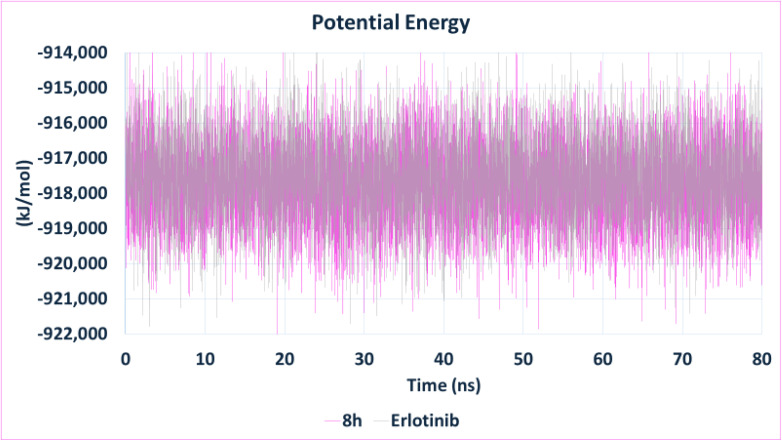
Potential energy profile of the EGFR–ligand complexes over the 80 ns simulation. Both compound 8h and erlotinib demonstrated thermodynamic stability with consistent potential energy values around −918 000 kJ mol^−1^, confirming well-equilibrated systems.

Finally, potential energy profiles (Fig. 16) confirmed the thermodynamic stability of both systems, with 8h exhibiting energy values comparable to those of erlotinib (∼−918 000 kJ mol^−1^) and consistent fluctuations. This reflects a well-minimized and equilibrated system under production conditions.

In summary, compound 8h displayed strong and persistent hydrogen bonding, favorable compactness, low residue fluctuation, and stable energy behavior, collectively demonstrating its robust dynamic stability in complex with EGFR. These results are in strong agreement with experimental IC_50_ data and reinforce the promise of compound 8h as a potent and stable EGFR inhibitor.

### 3.4. Quantum mechanical (QM) computations for compound 8h

Quantum mechanical (QM) calculations were employed to gain deeper insight into the electronic structure, reactivity, and interaction potential of compound 8h, the leading derivative in our series.^88^ These computations, comprising Density Functional Theory (DFT) and Molecular Electrostatic Potential (MEP) analyses, were performed to support and complement the findings from molecular docking, molecular dynamics (MD) simulations, and biological assays, providing a unified mechanistic view of its inhibitory action on EGFR and VEGFR-2.^89^

#### 3.4.1. Density functional theory (DFT) analysis of compound 8h

**Fig. 17:**
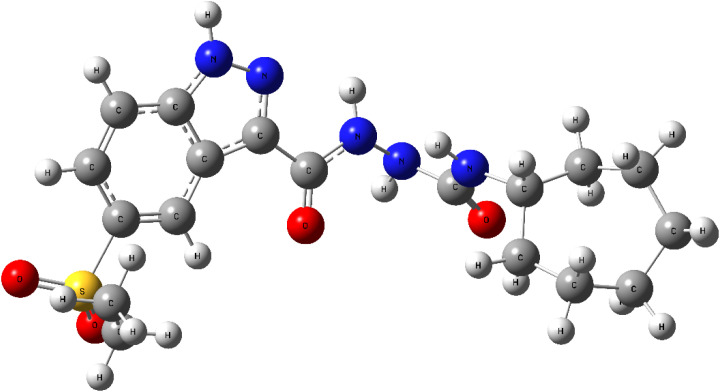
Optimized molecular geometry of compound 8h, calculated at the B3LYP/6-311+G(2d,p) level, confirming a stable conformation suitable for further electronic analysis.

The optimized molecular geometry of compound 8h (Fig. 17) was obtained using the B3LYP/6-311+G(2d,p) level of theory, and confirmed as a true minimum through frequency analysis.^90^

**Fig. 18:**
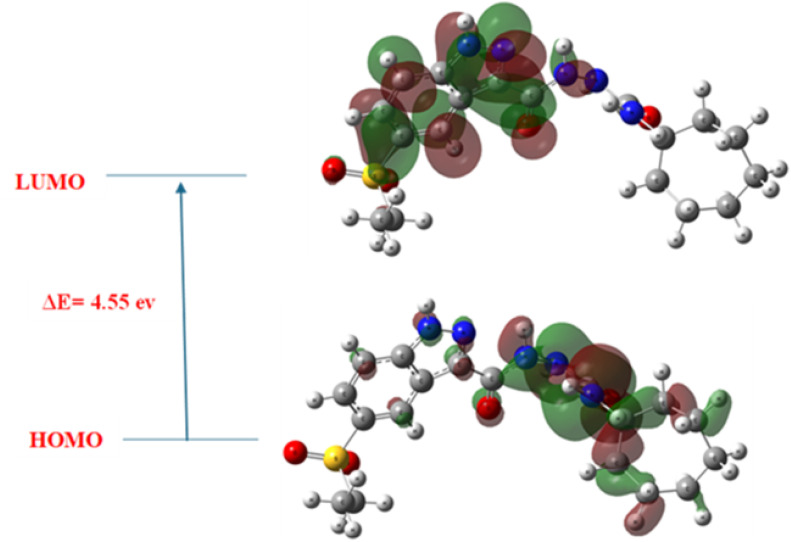
Frontier molecular orbitals (HOMO–LUMO) of compound 8h, with an energy gap (Δ*E*) of 4.55 eV. HOMO is delocalized over the indazole and urea regions, while LUMO extends toward the cycloheptyl moiety, indicating functional electronic partitioning relevant to binding.

The calculated HOMO–LUMO energy gap (Δ*E*) of 4.55 eV (Fig. 18) reflects a well-balanced electronic profile, indicating chemical stability with moderate reactivity—a desirable trait for bioactive drug candidates.

The corresponding chemical hardness (*η*) and softness (*σ*) values were found to be 2.28 eV and 0.44 eV,^1^ respectively. These parameters suggest that 8h possesses sufficient resistance to electronic deformation, yet remains adequately polarizable, enabling productive interactions with receptor binding pockets. Additionally, the calculated dipole moment of 9.3 debye highlights a pronounced molecular polarity, which enhances solubility and facilitates electrostatic interactions with key receptor residues crucial for maintaining binding specificity and orientation during dynamic simulations. This polarity also supports the sustained hydrogen bonding observed throughout MD trajectories, which distinguished 8h from reference inhibitors such as erlotinib.

The HOMO orbital was primarily localized on the urea linker and the terminal cycloheptyl region, indicating that these regions act as electron donors during receptor engagement. This is likely involved in π-stacking and hydrogen bonding with active-site residues, such as Met769 and Glu885, as supported by docking data. The LUMO orbital, on the other hand, extended toward the indazole ring, indicating a role in electron acceptance within the pocket, similar to that of Leu820 and Val702. This spatial electron distribution confirms the functional dichotomy of 8h, where the donor–acceptor pattern directly mirrors the observed binding orientation and interaction specificity as determined by docking studies.

#### 3.4.2. Molecular electrostatic potential (MEP) analysis

**Fig. 19:**
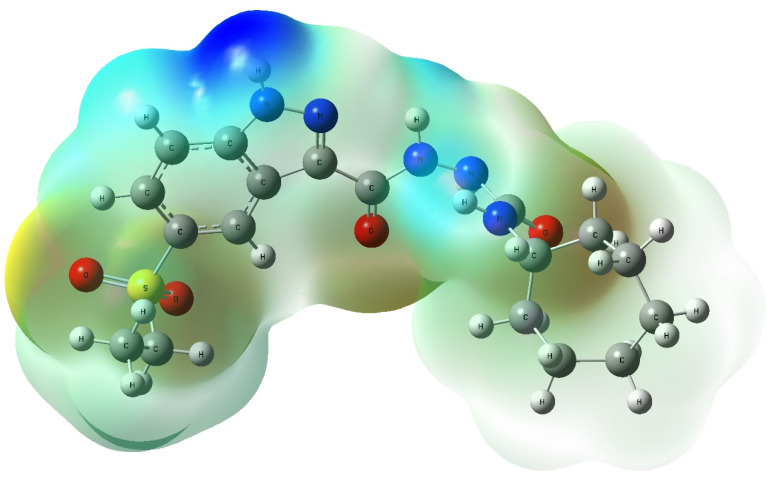
Molecular Electrostatic Potential (MEP) map of compound 8h, illustrating electron-rich (red/yellow) and electron-deficient (blue) regions. Key interaction sites correlate with residues observed in docking and MD simulations.

The MEP surface of compound 8h (Fig. 19) further visualizes the electrostatic potential landscape of the molecule, delineating nucleophilic (red and yellow) and electrophilic (blue) regions critical for ligand–protein recognition. Notably, highly electronegative zones were observed around the carbonyl and sulfonyl oxygen atoms, as well as the indazole nitrogen, consistent with their role as hydrogen bond acceptors during docking with EGFR (Met769, Lys721) and VEGFR-2 (Glu885, Asp1046). Conversely, electron-deficient sites around urea N–H groups and methylene hydrogens may act as hydrogen bond donors, complementing the polar environment of kinase binding pockets.

Collectively, the DFT-derived orbital distributions, reactivity descriptors (Δ*E*, *η*, *σ*), and MEP surface of compound 8h are in strong concordance with the outcomes of molecular docking and MD simulation. The alignment of high electron density with known interaction sites, along with its calculated polarity and frontier orbital behavior, reinforces the hypothesis that the pharmacophoric architecture of the indazole core, urea linker, and cycloheptyl tail is electronically and spatially optimized for dual kinase inhibition. These QM findings substantiate the mechanistic rationale behind the biological efficacy of 8h and offer a valuable framework for further structure-based optimization.

### 3.5. ADME studies

In the comprehensive assessment of compound 8h, ADME predictions provided critical insights into its pharmacokinetic behavior and drug-likeness, validating its potential as a promising dual EGFR/VEGFR-2 inhibitor. SwissADME analysis revealed that 8h complies fully with Lipinski's, Ghose's, and Muegge's filters, reflecting a favorable balance of physicochemical properties required for oral bioavailability. Despite a TPSA of 141.43 Å^2^, which slightly exceeds the thresholds of Veber and Egan rules, the compound still achieves a bioavailability score of 0.55, comparable to that of erlotinib, the clinical benchmark.

From a solubility perspective, compound 8h was classified as moderately soluble by multiple predictive models (ESOL, Ali, SILICOS-IT), which supports its feasibility for oral formulations with minimal solubilizing excipients. Although GI absorption is predicted to be low, this may be attributed to its relatively high polar surface area and the presence of four hydrogen bond donors, both of which can limit passive permeability. However, this drawback may be counterbalanced by formulation strategies or prodrug approaches. Notably, 8h is predicted to be a *P*-glycoprotein (*P*-gp) substrate, which may limit intracellular accumulation, but unlike erlotinib, it does not inhibit major CYP450 isoforms except CYP3A4. This is a crucial distinction: while erlotinib acts as a multi-CYP inhibitor (CYP1A2, CYP2C19, CYP2C9, CYP2D6, and CYP3A4), posing a higher risk for drug–drug interactions, 8h exhibits greater metabolic selectivity, suggesting a safer pharmacokinetic profile for clinical development.

In terms of lipophilicity, compound 8h displays a consensus log *P*_o/x_ of 2.08, reflecting a balanced hydrophilic–lipophilic profile suitable for receptor interaction and membrane diffusion. This contrasts with the higher log *P*_o/x_ of 3.20 for erlotinib, which, while advantageous for permeability, may contribute to off-target effects or poor aqueous solubility. The lower skin permeability (log *K*_p_ = −7.04 cm s^−1^) of 8h compared to erlotinib (−6.35 cm s^−1^) further supports its selectivity for non-dermal administration routes.

Complementing these ADME findings, molecular dynamics simulations showed that compound 8h maintains a stable complex with EGFR, characterized by consistent RMSD values and persistent hydrogen bonding throughout the simulation period. The QM calculations supported this behavior by demonstrating a HOMO–LUMO energy gap of 4.55 eV, a dipole moment of 9.3 debye, and an MEP surface rich in electron-dense regions conducive to binding. These electronic characteristics, along with structural features such as the indazole core and cycloheptyl tail, rationalize the strong docking affinity and inhibitory potency observed experimentally.

Taken together, the ADME profile of compound 8h complements and reinforces its biological activity, docking orientation, dynamic behavior, and electronic structure. While erlotinib maintains clinical efficacy, its broader CYP inhibition and BBB permeability pose limitations to its use. In contrast, 8h emerges as a more selective, synthetically accessible, and electronically optimized candidate, aligning well with modern drug design standards and offering a lower potential for off-target interactions. These integrated findings highlight 8h as a viable and rationally designed lead compound for further preclinical development in dual kinase inhibition strategies.

### 3.6. Structural–activity relationship (SAR) analysis

The following are some key points outlining the SAR of the newly synthesized compounds 8a–l.
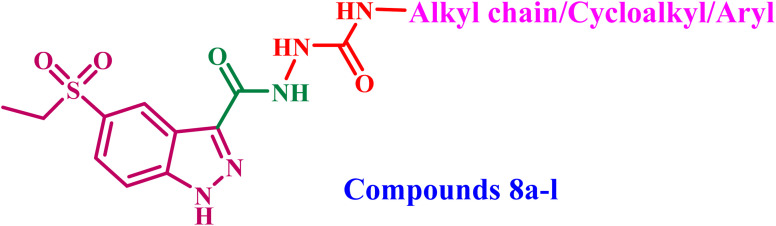


(1) *The indazole ring* is significantly embedded within the EGFR active site, establishing conventional hydrogen bonds with Met769 (hinge region), a critical interaction necessary for ATP-competitive inhibition. Moreover, the sulfonyl group of the indazole moiety in the VEGFR-2 active site is situated within the ATP-binding cleft, establishing a hydrogen bond with the His1026 residue, which is essential for inhibiting the VEGFR-2 kinase.

(2) *The urea linker* forms dual hydrogen bonds with Met769 and Pro770, providing both rigidity and directional orientation within the EGFR active site. In VEGFR-2, the urea linker binds the ligand through hydrogen bonding with the Glu885 and Asp1046 residues, thereby enhancing the spatial alignment required for optimal pocket occupancy.

(3) *The cycloheptyl tail* in VEGFR-2 extends into a hydrophobic sub-pocket, where it forms alkyl interactions with Cys919 and Leu1035, residues positioned around the outer edge of the binding cleft. In EGFR, the cycloheptyl tail, despite being solvent-exposed, augments binding enthalpy without compromising core interactions.

## 4. Conclusion

In conclusion, a new series of 5-ethylsulfonyl-indazole-3-carboxamides (8a–l) was synthesized and fully characterized. All compounds were tested *in vitro* for their antiproliferative activity against the breast cancer cell line (MCF-7) and the colorectal carcinoma cell line (HCT-116). The results showed that compounds 8g and 8h were the most potent derivatives against the two MCF-7 and HCT-116 cancer cell lines. Compounds 8g and 8h demonstrated a good safety margin against the normal human diploid cell line (WI-38) with IC_50_ values exceeding 120 nM. Furthermore, to confirm the mechanism of action as anticancer agents, the most potent antiproliferative derivatives, 8g and 8h, were further evaluated for their activity against VEGFR-2 and EGFR. The results indicated that compounds 8g and 8h exhibited significant VEGFR-2 inhibitory action (IC_50_ values of 16 and 12 nM, respectively) compared to sorafenib, as well as EGFR inhibition (IC_50_ values of 71 and 68 nM, respectively) compared to erlotinib. The correlation between structure and activity (SAR) was elaborated upon. Compounds 8g and 8h induced apoptosis by the activation of caspases-3, 8, 9, Bax, and p53, while inhibiting Bcl-2. Moreover, compared to dexamethasone (82.7 and 93.2%), compound 8h reduced the levels of the immunomodulatory proteins TNF-α by 82% and IL-6 by 91%. The comprehensive computational analysis of compound 8h offers strong theoretical validation for its experimentally observed dual inhibitory potency. Molecular docking confirmed favorable orientation and binding energy within both EGFR and VEGFR-2 kinase domains, while MD simulations highlighted the structural stability and sustained receptor engagement over time. QM studies further elucidated the compound's electronic suitability for receptor binding, with an ideal HOMO–LUMO energy gap and functional orbital localization supporting electrostatic complementarity. ADME predictions complemented these findings, indicating that 8h possesses favorable drug-likeness, synthetic accessibility, and reduced metabolic liability compared to standard EGFR inhibitors. Collectively, these data underscore the robust design and therapeutic promise, validating the integrated use of computational tools in guiding the development of dual kinase inhibitors.

## Conflicts of interest

The author disclosed there were no potential conflicts of interest.

## Funding

The authors acknowledge the support by Princess Nourah bint Abdulrahman University Researchers Supporting Project Number (PNURSP2025R3), Princess Nourah bint Abdulrahman University, Riyadh, Saudi Arabia. The authors also acknowledge support from the KIT-Publication Fund of the Karlsruhe Institute of Technology.

## Supplementary Material

RA-015-D5RA07017A-s001

## Data Availability

The authors declare that the data supporting the findings of this study are available within the supplementary information (SI). Supplementary information is available. See DOI: https://doi.org/10.1039/d5ra07017a.

## References

[cit1] Sung H., Ferlay J., Siegel R. L., Laversanne M., Soerjomataram I., Jemal A., Bray F. (2021). Ca-Cancer J. Clin..

[cit2] Al-Wahaibi L. H., Elshamsy A. M., Ali T. F., Youssif B. G., Bräse S., Abdel-Aziz M., El-Koussi N. A. (2024). ACS Omega.

[cit3] Al-Wahaibi L. H., Hafez H. M., Edrees F. H., Abou-Zied H. A., Youssif B. G., Bräse S. (2025). J. Mol. Struct..

[cit4] Yang Y., Li S., Wang Y., Zhao Y., Li Q. (2022). Signal Transduction Targeted Ther..

[cit5] Zhang N., Li Y. (2023). MedComm.

[cit6] Ghosh S., Marrocco I., Yarden Y. (2020). Adv. Cancer Res..

[cit7] Sudhesh Dev S., Zainal Abidin S. A., Farghadani R., Othman I., Naidu R. (2021). Front. Pharmacol.

[cit8] Rozen E. J., Shohet J. M. (2022). Cancer Metastasis Rev..

[cit9] Kaufman N. E., Dhingra S., Jois S. D., Vicente M. d. G. H. (2021). Molecules.

[cit10] Al-Wahaibi L. H., Mahmoud M. A., Mostafa Y. A., Raslan A. E., Youssif B. G. (2023). J. Enzyme Inhib. Med. Chem..

[cit11] El-Sherief H. A., Youssif B. G., Abdelazeem A. H., Abdel-Aziz M., Abdel-Rahman H. M. (2019). Anti-Cancer Agents Med. Chem..

[cit12] Al-Wahaibi L. H., El-Sheref E. M., Hassan A. A., Bräse S., Nieger M., Youssif B. G., Ibrahim M. A., Tawfeek H. N. (2023). Pharmaceuticals.

[cit13] Grobbelaar C., Steenkamp V., Mabeta P. (2025). Curr. Issues Mol. Biol..

[cit14] Marzouk A. A., Abdel-Aziz S. A., Abdelrahman K. S., Wanas A. S., Gouda A. M., Youssif B. G., Abdel-Aziz M. (2020). Bioorg. Chem..

[cit15] Shah A. A., Kamal M. A., Akhtar S. (2021). Curr. Drug Metab..

[cit16] Tabernero J. (2007). Mol. Cancer Res..

[cit17] Hu L., Fan M., Shi S., Song X., Wang F., He H., Qi B. (2022). Eur. J. Med. Chem..

[cit18] Bojovic D., Nikolic M., Nedeljkovic N., Vesovic M., Zivanovic A., Karovic M. (2025). Chem. Biodiversity.

[cit19] Pearce S. , Drug Discovery, 2017, p. 67

[cit20] Kumar A., Mishra A. (2023). J. Exp. Zool..

[cit21] Vitaku E., Smith D. T., Njardarson J. T. (2014). J. Med. Chem..

[cit22] Rusu A., Moga I.-M., Uncu L., Hancu G. (2023). Pharmaceutics.

[cit23] Adhav V. A., Saikrishnan K. (2023). ACS Omega.

[cit24] Thomas N. M., Alharbi M., Muripiti V., Banothu J. (2025). Mol. Diversity.

[cit25] Gmeiner W. H., Okechukwu C. C. (2023). Cancer Drug Resist..

[cit26] Tan C., Yang S.-J., Zhao D.-H., Li J., Yin L.-Q. (2022). Arabian J. Chem..

[cit27] Mérour J.-Y., Buron F., Plé K., Bonnet P., Routier S. (2014). Molecules.

[cit28] Bhane P. D., Pawar S. S. (2025). Med. Chem..

[cit29] Boddapati S. M., Chalapaka B., Kola A. E., Jonnalagadda S. B. (2025). Top. Curr. Chem..

[cit30] Bhat U. V., Martis G. J., Chikkanna D., Gaonkar S. L. (2025). J. Chem..

[cit31] Evren A. E., Kaya A. Z., Karakaya A., Tutuş B., Güngör E. M., Nuha D., Osmaniye D., Kaya B., Çevik U. A., Yurttaş L. (2025). Future Med. Chem..

[cit32] Tandon R., Singh I., Luxami V., Tandon N., Paul K. (2019). Chem. Rec..

[cit33] Engel J., Richters A., Getlik M. u., Tomassi S., Keul M., Termathe M., Lategahn J., Becker C., Mayer-Wrangowski S., Grütter C. (2015). J. Med. Chem..

[cit34] Frejat F. O. A., Zhai H., Cao Y., Wang L., Mostafa Y. A., Gomaa H. A., Youssif B. G., Wu C. (2022). Bioorg. Chem..

[cit35] Qi H., Chen L., Liu B., Wang X., Long L., Liu D. (2014). Bioorg. Med. Chem. Lett..

[cit36] Elsayed N. M., Abou El Ella D. A., Serya R. A., Tolba M. F., Shalaby R., Abouzid K. A. (2016). MedChemComm.

[cit37] Xu Y., Dang R., Guan J., Xu Z., Zhao S., Hu Y. (2018). J. Heterocycl. Chem..

[cit38] Sazeli S., Nath A. R., Ahmad M. H., Zulkifli N., Johan M. R., Yehye W. A., Voon L. H. (2021). RSC Adv..

[cit39] Prakash C. R., Raja S., Saravanan G. (2012). Chin. Chem. Lett..

[cit40] Perković I., Butula I., Kralj M., Martin-Kleiner I., Balzarini J., Hadjipavlou-Litina D., Katsori A.-M., Zorc B. (2012). Eur. J. Med. Chem..

[cit41] Eissa I. H., Ibrahim M. K., Alesawy M. S., El-Adl K. (2022). Arch. Pharm..

[cit42] Hu H., Huang J., Cao Y., Zhang Z., He F., Lin X., Wu Q., Zhao S. (2022). Molecules.

[cit43] Wheler J. J., Janku F., Naing A., Li Y., Stephen B., Zinner R., Subbiah V., Fu S., Karp D., Falchook G. S. (2016). Mol. Cancer Ther..

[cit44] Irannejadrankouhi S., Mivehchi H., Eskandari-Yaghbastlo A., Nejati S. T., Emrahoglu S., Azarang F., Nikroo A., Nabi-Afjadi M. (2025). Pharmacol. Rep..

[cit45] Yao S.-X., Huang Y.-J., Zhang Y.-X., Cui Z.-X., Lu H.-Y., Wang R., Shi L. (2025). J. Drug Targeting.

[cit46] Yousefbeyk F., Ghasemi S. (2025). Pharm. Sci..

[cit47] Mahmoud M. A., Mohammed A. F., Salem O. I., Almutairi T. M., Bräse S., Youssif B. G. (2024). J. Enzyme Inhib. Med. Chem..

[cit48] Mahmoud M. A., Mohammed A. F., Salem O. I., Rabea S. M., Youssif B. G. (2023). J. Mol. Struct..

[cit49] Mostafa Y. A., Assoud J. A., Desoky A. Y., Mohamady S., Mohamed N. M., Salem O. I., Almarhoon Z. M., Bräse S., Youssif B. G. (2024). Front. Chem..

[cit50] Elwan A. , Mabrouk R. R., Musa A., Saleh Al Ward M. M., Hussein S., Awaji A. A. and El-Zahabi M. A., Synthesis, Molecular Docking, and Anti-Proliferative Evaluation, 2025 10.1039/d5ra03829d PMC12377202 40860085

[cit51] Eissa I. H., El-Haggar R., Dahab M. A., Ahmed M. F., Mahdy H. A., Alsantali R. I., Elwan A., Masurier N., Fatahala S. S. (2022). J. Enzyme Inhib. Med. Chem..

[cit52] Marques C. S., Brandão P., Burke A. J. (2024). Molecules.

[cit53] Ramadan M., Abd El-Aziz M., Elshaier Y. A., Youssif B. G., Brown A. B., Fathy H. M., Aly A. A. (2020). Bioorg. Chem..

[cit54] Alshammari M. B., Aly A. A., Youssif B. G., Bräse S., Ahmad A., Brown A. B., Ibrahim M. A., Mohamed A. H. (2022). Front. Chem..

[cit55] Al-Wahaibi L. H., Mostafa Y. A., Abdelrahman M. H., El-Bahrawy A. H., Trembleau L., Youssif B. G. (2022). Pharmaceuticals.

[cit56] Youssif B. G., Mohamed A. M., Osman E. E. A., Abou-Ghadir O. F., Elnaggar D. H., Abdelrahman M. H., Treamblu L., Gomaa H. A. (2019). Eur. J. Med. Chem..

[cit57] Chandler D. P., Wagnon C. A., Bolton Jr H. (1998). Appl. Environ. Microbiol..

[cit58] Lee J.-C., Chow N.-H., Wang S.-T., Huang S.-M. (2000). Eur. J. Cancer.

[cit59] Hlatky L., Tsionou C., Hahnfeldt P., Coleman C. N. (1994). Cancer Res..

[cit60] Plati J., Bucur O., Khosravi-Far R. (2008). J. Cell. Biochem..

[cit61] Dewa W., Handharyani E., Purawaningsih S., Mariya S. (2024). Adv. Anim. Vet. Sci..

[cit62] Shaik M. R., Kandaswamy K., Guru A., Khan H., Giri J., Mallik S., Shah M. A., Arockiaraj J. (2024). BMC Oral Health.

[cit63] Altaf I. , Jan N., Sofi S. and Mir M. A., in p53 in Breast Cancer, CRC Press, 2025, pp. 1–19

[cit64] Wu X., Gu R., Tang M., Mu X., He W., Nie X. (2025). Burns Trauma.

[cit65] Kouwenhoven W. M., Robinson E. J., Hamberg D., von Oerthel L., Smidt M. P., van der Heide L. P. (2025). Cell Death Dis..

[cit66] Mohassab A. M., Hassan H. A., Abou-Zied H. A., Fujita M., Otsuka M., Gomaa H. A., Youssif B. G., Abdel-Aziz M. (2024). J. Mol. Struct..

[cit67] Dunlop R. J., Campbell C. W. (2000). J. Pain Symptom Manage..

[cit68] Zaporowska-Stachowiak I., Springer M., Stachowiak K., Oduah M., Sopata M., Wieczorowska-Tobis K., Bryl W. (2024). J. Interferon Cytokine Res..

[cit69] Shang G.-S., Liu L., Qin Y.-W. (2017). Oncol. Lett..

[cit70] Balkwill F. (2006). Cancer Metastasis Rev..

[cit71] Sousa S. F., Ribeiro A. J., Neves R. P., Brás N. F., Cerqueira N. M., Fernandes P. A., Ramos M. J. (2017). Wiley Interdiscip. Rev.: Comput. Mol. Sci..

[cit72] Guedes I. A., Pereira F. S., Dardenne L. E. (2018). Front. Pharmacol.

[cit73] Shukla R., Tripathi T. (2020). Comput.-Aided Drug Des..

[cit74] Naqvi A. A., Mohammad T., Hasan G. M., Hassan M. I. (2018). Curr. Top. Med. Chem..

[cit75] Velankar S. , Burley S. K., Kurisu G., Hoch J. C. and Markley J. L., Structural Proteomics: High-Throughput Methods, 2021, pp. 3–21

[cit76] Al-Wahaibi L. H., Abou-Zied H. A., Mahmoud M. A., Youssif B. G., Bräse S., Rabea S. M. (2025). J. Enzyme Inhib. Med. Chem..

[cit77] Luo D., Tong J.-B., Zhang X., Xiao X.-C., Bian S. (2022). J. Mol. Struct..

[cit78] Khan M. N., Farooq U., Khushal A., Wani T. A., Zargar S., Khan S. (2025). PLoS One.

[cit79] Modi S. J., Kulkarni V. M. (2022). J. Biomol. Struct. Dyn..

[cit80] Baba H. , Bouqdayr M., Jouimyi M. R., Elmessaoudi-Idrissi M. and Kettani A., A Simple Overview for Proteins Molecular Dynamics Simulations Using GROMACS, International Conference on Advanced Intelligent Systems for Sustainable Development, Springer Nature, Cham, Switzerland, 2023, pp. 355–363

[cit81] Croitoru A., Kumar A., Lambry J.-C., Lee J., Sharif S., Yu W., MacKerell Jr A. D., Aleksandrov A. (2025). J. Chem. Theory Comput..

[cit82] Wang L., O'Mara M. L. (2021). J. Chem. Theory Comput..

[cit83] Emperador A., Crehuet R., Guàrdia E. (2021). Polymers.

[cit84] Koneru J. K. , Reid K. M. and Robustelli P., arXiv, 2025, preprint, arXiv:2505.01860, 10.48550/arXiv.2505.01860

[cit85] Ke Q., Gong X., Liao S., Duan C., Li L. (2022). J. Mol. Liq..

[cit86] Fábián B., Thallmair S., Hummer G. (2023). J. Chem. Theory Comput..

[cit87] Simmonett A. C., Brooks B. R. (2021). J. Chem. Phys..

[cit88] Ganiev B., Mardonov U., Kholikova G. (2023). Mater. Today: Proc..

[cit89] UrRehman S., Anwer M., BiBi S., Jamil S., Yasin M., Khan S. R., Nadeem R., Ali S., Jia R. (2022). Mater. Sci. Semicond. Process..

[cit90] Gray M., Bowling P. E., Herbert J. M. (2024). J. Phys. Chem. A.

